# Regulation of the antennal transcriptome of the dengue vector, *Aedes aegypti,* during the first gonotrophic cycle

**DOI:** 10.1186/s12864-020-07336-w

**Published:** 2021-01-21

**Authors:** Sharon Rose Hill, Tanvi Taparia, Rickard Ignell

**Affiliations:** 1 Disease Vector Group, Department of Plant Protection Biology, Swedish University of Agricultural Sciences, 230 54 Alnarp, Sweden; 2 Business Unit Biointeractions and Plant Health, Wageningen University and Research, AA 6700 Wageningen, The Netherlands

**Keywords:** Mosquito, Olfaction, Ontogeny, Chemosensory-related genes, Neuromodulatory genes, Transcription factors

## Abstract

**Background:**

In the light of dengue being the fastest growing transmissible disease, there is a dire need to identify the mechanisms regulating the behaviour of the main vector *Aedes aegypti*. Disease transmission requires the female mosquito to acquire the pathogen from a blood meal during one gonotrophic cycle, and to pass it on in the next, and the capacity of the vector to maintain the disease relies on a sustained mosquito population.

**Results:**

Using a comprehensive transcriptomic approach, we provide insight into the regulation of the odour-mediated host- and oviposition-seeking behaviours throughout the first gonotrophic cycle. We provide clear evidence that the age and state of the female affects antennal transcription differentially. Notably, the temporal- and state-dependent patterns of differential transcript abundance of chemosensory and neuromodulatory genes extends across families, and appears to be linked to concerted differential modulation by subsets of transcription factors.

**Conclusions:**

By identifying these regulatory pathways, we provide a substrate for future studies targeting subsets of genes across disparate families involved in generating key vector behaviours, with the goal to develop novel vector control tools.

**Supplementary Information:**

The online version contains supplementary material available at 10.1186/s12864-020-07336-w.

## Background

More than 80% of the world’s population is at risk of contracting a vector-borne disease, accounting for more than 17% of all infectious diseases worldwide, and causing ca. 700,000 deaths annually [1]. As the primary vector of arboviral diseases, including dengue, Zika, chikungunya and yellow fever, the mosquito *Aedes aegypti* accounts for ca. 140 million diagnosed cases of infections annually [1]. The capacity of female mosquitoes to vector these diseases is directly dependent on females locating a suitable host and taking a complete blood meal, behaviours greatly influenced by, e.g. age and nutritional status [2–4]. Throughout the life cycle of the female mosquito, these vector-related behaviours are regulated by internal factors and sensory input, predominantly derived from olfactory cues [2, 3]. Characterising the molecular apparatus that mediates the peripheral detection of odorants, throughout the gonotrophic cycle, will improve our understanding of the dynamic nature of the peripheral olfactory system of female mosquitoes, and may provide targets for use in novel vector monitoring and control strategies.

**Fig. 1:**
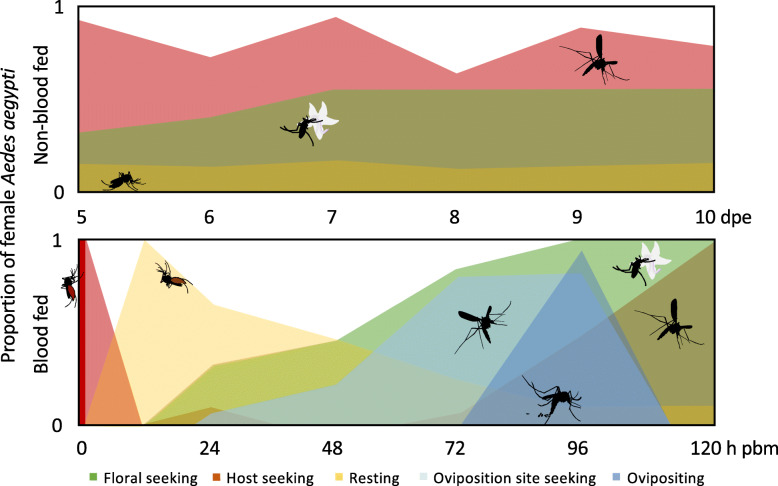
Schematic representation of the gonotrophic cycle of *Aedes aegypti* females. After adult maturation, non-blood fed mosquitoes share their time amongst floral seeking [6, 7], host seeking [8–10] and resting ([6] and refs therein) (top panel). Following a complete blood meal at 5 days post-emergence (dpe), the host seeking behaviour is inhibited until egg-laying [11–13], while floral seeking is inhibited for up to 48 h [7, 13], when pre-oviposition behaviours commence [14] (bottom panel). Most females have oviposited within 100 h post-blood meal (pbm) [15]

The first gonotrophic cycle of a female *Ae. aegypti* succeeds the approximately 5-day long adult maturation and mating period [5] (Fig. 1). During this period, females engage in active host seeking, which continues until the female, with sufficient energetic reserves, takes a complete blood meal [5]. While these behaviours are often considered stereotypic, the dynamic nature of host seeking and blood feeding has been demonstrated over the first 2 weeks post-emergence [8–10, 16, 17]. A stronger dynamic change in these behaviours is demonstrated immediately following a successful blood meal when females locate a resting site, reduce flight activity and demonstrate refractoriness to host odours [11–13]. Blood meal digestion and egg development continues for up to 60 h, and is followed by gravid females displaying pre-oviposition behaviour, i.e. the search for suitable egg-laying sites [5]. Oviposition usually occurs a few hours after the completion of egg maturation, around 96 h post-blood meal (pbm) [5, 18], at which time the host odour refractoriness is lifted and then host seeking resumes within 24 h [19].

Expression profiling of chemosensory-related genes in the main olfactory organ, the antenna, throughout the gonotrophic cycle of the female mosquito can quantify, and thus provide insights into the regulation of the molecular correlates of the various olfactory-driven, and vector-related, behaviours [17, 20–23]. Previous gene expression analyses have described the genetic regulation of the peripheral olfactory system of female mosquitoes during defined periods associated with behavioural change, including maturation [17, 24, 25], post-blood meal olfactory refractoriness [11, 19–23] and pre-oviposition behaviour [21]. These studies collectively show that differential gene abundance is linked with age- and/or state-dependant concerted changes in both sensory and behavioural sensitivity to resource-related odours [17, 20–25].

The objective of this study is to perform a comprehensive analysis, throughout the first gonotrophic cycle, of genes involved in the regulation of the peripheral olfactory system of age-matched host-seeking and blood-fed female *Ae. aegypti*. This study explores several gene families directly involved in chemosensation or its regulation, including the chemoreceptors, binding proteins, modulators and their cognate receptors, enzymes, transcription factors and circadian regulators. The putative role of these genes in odour detection and their correlation with the physiological state of the mosquito during aging and throughout the reproductive cycle is discussed. The future functional characterisation of the identified genes and how they regulate gonotrophic behaviours may provide targets for use in future vector control methods.

## Results

### Global gene expression profiling

Expression profiling of antennal mRNA from the 36 libraries created at six time points from the gonotrophic cycle of *Ae*. *aegypti*, revealed the reliable expression of 11,751 genes above background levels, of which 8579 genes were reliably detected in all libraries, while 9015 and 9245 genes were reliably detected in the non-blood fed (nbf) and blood fed (bf) libraries, respectively.

#### Controlled time for dissection allows for age comparison of gene expression profiles

To assess the efficacy of the narrow time window of tissue collection each day, the abundance of the six circadian clock transcripts, *period* (*PER*), *cycle* (*cyc*), *timeless* (AAEL019461), *clock* (AAEL022593), *vrille* (AAEL011371) and *par-domain protein-1* (*PDP1*) was analysed in the context of the diel patterns previously described [26–28]. Since the variation in transcript abundance over time amongst the clock genes was demonstrated to be low, and was not accentuated in the anticycling genes e.g. *Clock* and *PDP1*, the variation is likely not due to diel or circadian effects (Fig. S1 insets). In fact, the observed patterns of abundance over time were consistent between *Clock* and *PDP1*, as well as between *PER*, *timeless* and *vrille* (Fig. S1). Thus, the changing abundance of the clock genes, over time, is likely more a result of age than diel or circadian rhythms.

#### Effect of age on gene expression profiles

A gene ontology (GO) analysis of the molecular function of genes reliably detected in the antennae of nbf females every 24 h from 5 to 10 days post-emergence (dpe) indicates that the overall proportion of these genes in each molecular function category remains consistent through time (Fig. S2). The molecular functions that described > 85% of the genes expressed in host-seeking adult female antenna were protein binding (GO:0005515), ribosome structural constituent (GO:0003735), oxidoreductase activity (GO:0016491), hydrolase activity (GO:0016787) and odorant binding (GO:0005549; Fig. S2).

**Fig. 2:**
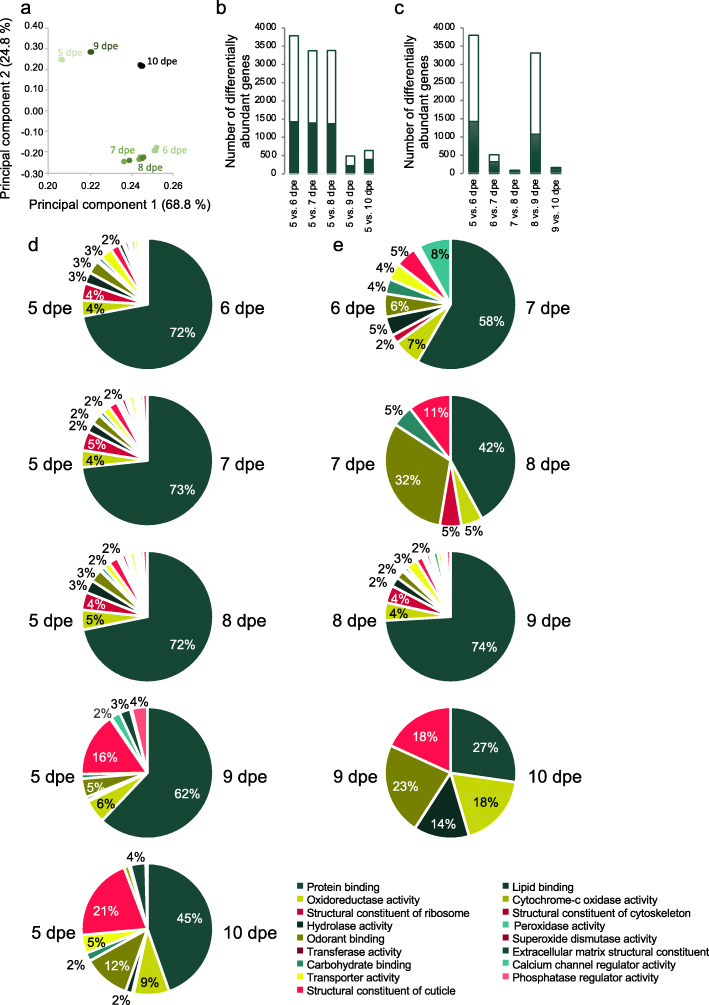
Age-dependent antennal transcript abundance. **a** Principal component analysis of the antennal transcriptomes of 5 to 10 days post-emergence (dpe) female *Aedes aegypti*. Ages are denoted by a gradient of green hues, with the lightest being 5 dpe and the darkest being 10 dpe. The total number of genes with differentially abundant transcripts from comparisons between **b** each age group and 5 dpe, and **c** adjacent ages of host-seeking adult female *Ae. aegypti* can be determined by the sum of those with gene ontology (GO) annotation (white) and those without (green). **d-e** Proportions of genes with differentially abundant transcripts in the antennae of 5 to 10 dpe host-seeking adult female *Ae. aegypti* classified by a level 3 molecular function gene ontology. Comparisons are made between each age group and 5 dpe (**d**), and adjacent age groups (**e**). The legend indicates the GO terms representing ≥2% of the total differentially abundant transcripts in at least one pairwise comparison

An overall comparison by principal component analysis (PCA) among the antennal transcriptomes from host-seeking females at each of the six ages revealed that age affected the transcript abundance (Fig. 2a). The replicates of each age clustered together, and there was no discernible difference among the antennal transcriptomes of ages 7 and 8 dpe (Fig. 2a). The transcriptomes demonstrated age-dependent oscillations along principal component axes 1 and 2 (Fig. 2a). Transcriptomes which align with each other on the principal component 2 axis (i.e. 5, 9 and 10 dpe or 6, 7 and 8 dpe) revealed fewer differentially abundant genes when compared with each other, as compared to those separated along this axis (e.g. Fig. 2b, c), indicating a change from one state of overall gene expression in the antennae of host-seeking females to another between 5 and 6 dpe, and then a return to the initial 5 dpe-like state between 8 and 9 dpe (Fig. 2a, b, c).

A comparison of the number of differentially abundant genes in the antennae of host-seeking females supported the findings from the PCA by demonstrating the largest differences between 5 and 6 dpe, followed by those between 8 and 9 dpe (Fig. 2c). Moreover, a careful examination of the genes differentially expressed between 5 dpe and 9 dpe revealed that 71% of the differentially expressed genes are shared between the 5 to 6 dpe and the 8 to 9 dpe comparisons. Of these 2657 shared genes, more than 99% were counter-regulated at these two time points, i.e., up-regulated at 6 dpe and down-regulated at 9 dpe (1401 genes), or vice versa (1235 genes). Indeed, more than 99% of the differentially abundant genes involved in regulating transcription were up-regulated between 5 and 6 dpe, and then down-regulated between 8 and 9 dpe. The relatively few differentially regulated genes evident among the antennae of either the 5, 9 and 10 dpe, or the 6, 7 and 8 dpe females (Fig. 2b, c), and the large number of genes counter-regulated between 5 to 6 dpe and 8 to 9 dpe, suggests that 5 dpe may represent the base state of antennal gene expression for a host-seeking female, established at the end of maturation. The base state appears to undergo a general, age-dependent regulation of the antennal transcriptome to an alternate state by 6 dpe, which is maintained from 6 to 8 dpe, and then reverts to the base state at 9 dpe and maintained through 10 dpe (Fig. 2a, b, c).

The predominant molecular functional classes of the genes demonstrating age-dependent differentially abundant transcripts (Fig. 2d, e) reflected those of the most abundant classes, protein binding (GO:0005515), structural constituent of the ribosome (GO:0003735), oxidoreductase activity (GO:0016491), hydrolase activity (GO:0016787) and odorant binding (GO:0005549; Figs 2d, e and S3). It is important to note that while the pairwise comparisons between ages of the same state contain relatively few differentially abundant genes, the predominant molecular classes represented are generally the same as those listed above. The exceptions are the lack of differentially abundant hydrolases between 7 and 8 dpe, and structural constituents of ribosomes between 9 and 10 dpe. Each of these molecular functional classes are involved in the active regulation of the cellular environment in the antenna, be it by de novo synthesis and interaction of proteins with other proteins and/or ligands, or by the degradation of cell products and xenobiotics.

#### Effect of a blood meal on gene expression profiles

**Fig. 3:**
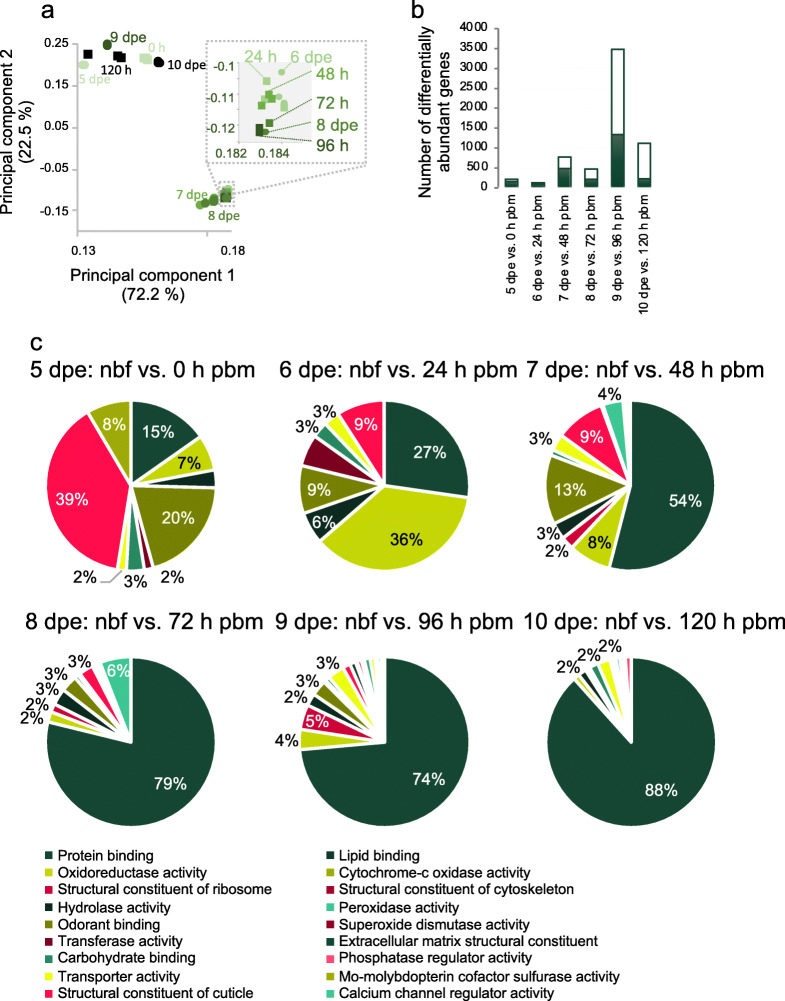
Age- and state-dependent antennal transcript abundance. **a** Principal component analysis of the antennal transcriptomes of non-blood fed (nbf; circles) and blood fed (bf; squares) female *Aedes aegypti*, 5 to 10 days post-emergence (dpe). Females were blood fed 5 dpe and the time is represented as hours post-blood meal (pbm). Ages are denoted by a gradient of green hues, with the lightest being 5 dpe and the darkest being 10 dpe. Inset: The area bordered by dotted grey lines is expanded for disambiguation. Three replicates of each antennal transcriptome for nbf and bf are depicted for each age. **b** Total number of genes with differentially abundant transcripts between the antennal transcriptomes of nbf and bf from 5 to 10 dpe adult female *Ae. aegypti* can be determined by the sum of those with gene ontology (GO) annotation (white) and those without (green). **c** Proportions of genes with differentially abundant transcripts in the antennae of age-matched host-seeking (nbf) and blood-fed (h pbm) adult female *Ae. aegypti* between 5 to 10 days post-emergence (dpe) were classified by a level 3 molecular function GO. The legend indicates the GO terms representing ≥2% of the total differentially abundant transcripts in at least one pairwise comparison

When comparing the antennal transcriptomes of nbf to bf age-matched cohorts, age accounted for more of the variation described by the principal component analysis than blood meal status, primarily on the principal component 2 axis (Fig. 3a). An exception to this was the antennal transcriptomes of females at 9 dpe, in which the antennal transcriptomes of nbf females and females 96 h pbm are not adjacent to each other on the principal component 2 axis, as predicted (Fig. 3a). Blood meal status was better described in the variation along the principal component 2 axis (Fig. 3a). Pairwise comparisons were not made between the genes expressed in the antennae of nbf 5 dpe females and those of the antennae from 6 to 10 dpe bf females, as has been done in previous studies (e.g. [21]), however an example of this is provided in the supplementary files for comparison (Fig. S3).

There were no genes exclusively and permanently turned on or off in the antenna in response to a blood meal during the first gonotrophic cycle. The largest number of differentially abundant genes between the antennal transcriptomes of nbf and bf females was found at 9 dpe, 96 h pbm, within 12±6 h of oviposition, followed by those at 10 dpe, 120 h pbm, post-oviposition (Fig. 3b). The fewest differentially abundant genes were identified in the antennae of 6 dpe, 24 h pbm, females (Fig. 3b). Immediately following a blood meal, the predominant molecular functions that were regulated at gene level were protein binding (GO:0005515), structural constituent of the cuticle (GO:0042302) and odorant binding (GO:0005549), while 24 h pbm oxidoreductase activity (GO:0016491) takes precedence (Fig. 3c). As the female progresses through the first gonotrophic cycle, these molecular functions remain predominant, however, the proportion of differentially abundant transcripts for protein binding increased at a constant rate (R^2^ = 0.91), while the others decrease proportionately (Fig. 3c). Within 1 h of the blood meal given at 5 dpe, regulation of cuticle constituent, odorant binding, and protein binding genes has commenced, however the genes regulating translation (GO:0003735) were not yet shown to be differentially abundant until 48 h pbm (Fig. 3c).

### Regulation of peripheral chemosensory genes

**Fig. 4:**
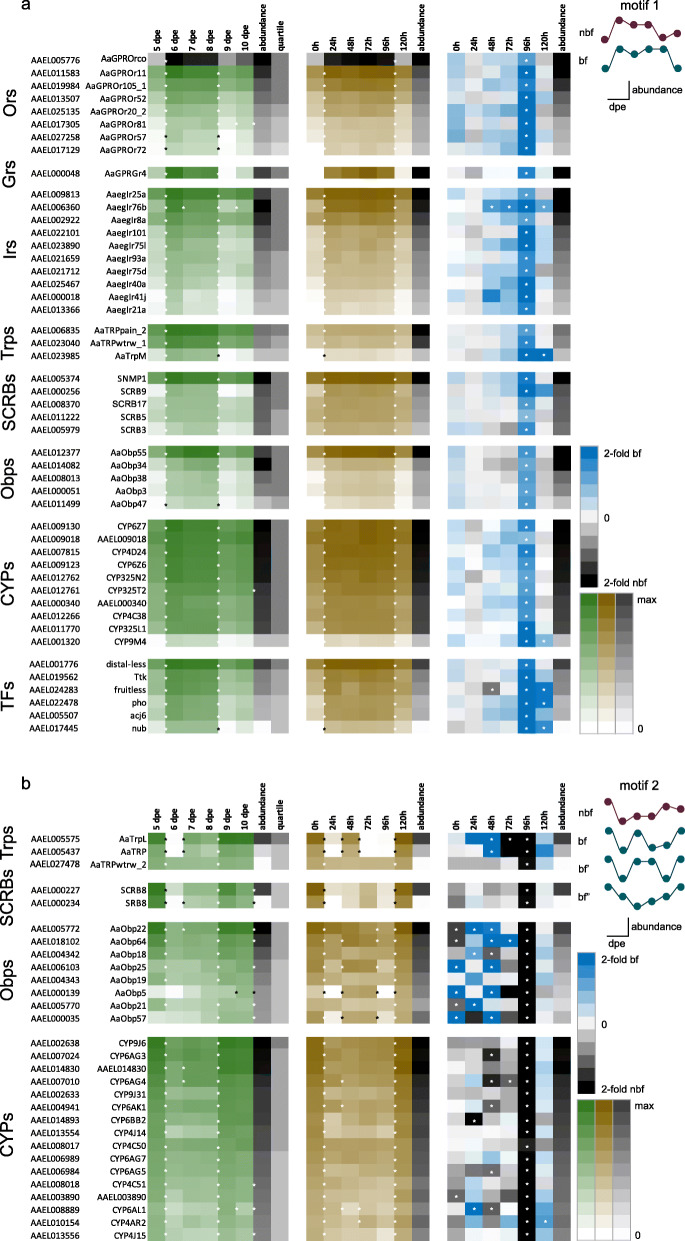
Concerted age- and state-dependent antennal gene regulation spanning gene families. Two regulation motifs (**a** and **b**) are demonstrated by the transcript abundance in 5 to 10 days post-emergence (dpe) non-blood fed (nbf; green; left) and age-matched blood fed (bf; brown; middle) *Aedes aegypti* female antennae. Comparisons between nbf (black) and age-matched bf (blue) antennal transcript abundance are described by fold change (right). Permanent gene identifiers along with the common gene names are to the left. Ball and stick diagrams represent the general trend in abundance demonstrated by this gene family (**a **motif 1, top right; **b **motif 2, top right). Asterisks between two age groups denote stringent significant difference (> 2-fold change; FDR *P* < 0.05). Asterisks to the far right of each table indicate significant differences between 5 and 10 dpe.  **a** These genes demonstrate a motif 1 pattern of transcript regulation, while **b** are those that demonstrate motif 2

Two motifs of concerted regulation were described for the chemosensory-related gene families. The overall trend in chemosensory-related gene abundance denoted as motif 1 was described by an increase with age between 5 and 6 dpe in nbf (Fig. 4 left; Figs. S4, S5, S6, S7, S8 and S9) and bf (Fig. 4 middle; Figs. S4, S5, S6, S7, S8 and S9) female antennae, although this was generally less pronounced post-blood meal. This overall high abundance was maintained until 9 dpe in nbf antennae (Fig. 4 left; Figs. S4, S5, S6, S7, S8 and S9), and until 10 dpe in bf antennae (Fig. 4 middle; Figs. S4, S5, S6, S7, S8 and S9), at which time it decreased to levels generally not significantly different from those of 5 dpe females (Fig. 4 right; Fig. S4). Motif 2 describes a similar, but inverted, trend in abundance in which abundance is down-regulated between 5 and 6 dpe in the antennae of nbf and bf females (Fig. 4 left; Figs. S4, S5, S6, S7, S8 and S9), and up-regulated in the antennae of nbf 9 dpe (Fig. 4 left; Figs. S4, S5, S6, S7, S8 and S9) and bf 10 dpe (Fig. 4 middle; Figs. S4, S5, S6, S7, S8 and S9) females. Of the two abundance motifs described in this study, odorant receptor (*Or)*, ionotropic receptor (*Ir)*, and class B scavenger receptor membrane bound protein (*SCRB)* overall gene regulation was described by motif 1, while the other chemosensory-related gene families were also described by motif 2, with genes that had an overall higher abundance tending to display motif 1, while those with lower abundance displayed motif 2. Comparisons that are mentioned below as being up- or down-regulated, or as differentially abundant, have significantly changed in abundance at least 2-fold (FDR *p* < 0.05), unless otherwise stated.

#### Odorant receptors

Of the repertoire of 97 annotated *Ors*, 86 and 87 were reliably detected in the antenna of nbf and bf adult females of *Ae. aegypti*, respectively, with a total of 90 when all ages and both feeding states are included (Fig. S4; Dataset S1). *Orco*, the gene encoding the obligate Or co-receptor [29], demonstrated the highest transcript abundance across all time points (Fig. 4; Fig. S4), amounting to an abundance similar to that of the unique *Ors* combined (Dataset S1). Motif 1 described the overall trend in *Or* abundance (Fig. 4), including both unique *Ors* and *Orco*, with the 24 h delay between 9 and 10 dpe in the antennae of bf female in returning to abundance levels similar to 5 dpe described by almost half of *Ors*, with a significantly higher abundance (> 2-fold; FDR *p* < 0.05) in bf compared with nbf antennae at 9 dpe (Fig. 4 a right; Fig. S4).

While many *Ors* appear to follow the motif 1 pattern of regulation (Fig. 4 a; Fig. S4), 19 *Ors* were not age- or state-dependently regulated, and several more *Ors* (e.g. *Or6*, *Or20_1* and *Or117*) exhibited a more variable pattern of abundance with age and reproductive status (Fig. S4). In a comparison of the abundance of antennal *Ors* from the oldest females tested (10 dpe) with the youngest (5 dpe), all of the 19 *Ors* identified exhibited significantly higher abundance in the older females, and all but two (*Or47* and *Or79*) also demonstrated a significant increase in abundance in the antennae of 6 dpe over 5 dpe females (Fig. S4 left). Following a blood meal, and controlling for age, 45 of the reliably detected *Ors* were not regulated compared with nbf (Fig. S4 right). Of the 44 regulated *Ors*, 39 were more abundant in the antennae of 96 h pbm females compared to non-blood fed females of the same age (9 dpe), while the other five (i.e., *Or20_1*, *Or25*, *Or42*, *Or79*, and *Or116*) were not regulated at this time point (Fig. 4 right; Fig. S4 right). Eleven of the *Ors* that demonstrated a higher abundance in the antennae 96 h pbm also displayed higher *Or* abundance at other times post-blood meal. Of particular interest, *Or117* was more significantly abundant in the antennae of females from 24 h to 96 h pbm, while *Or107*, and *Or13* and *Or20_2*, were significantly more abundant from 48 h and 72 h to 96 h, respectively (Fig. S7 right). Post-oviposition (120 h pbm), the level of abundance returned to that which was not significantly different from its age-matched cohort for all but two *Or*s, *Or79* and *Or105_2*, which were more abundant in nbf and bf antennae, respectively (120 h pbm; Fig. S4 right).

#### Gustatory receptors

The gustatory receptors were the least abundant chemosensory transcripts in the antennae (≤9.5 TPM per gene), with only five *Grs* reliably detected in the antennae (Fig. S5A). The pattern in abundance over time and post-blood meal described by *Gr4* is similar to that observed for the majority of the regulated *Ors*, exhibiting motif 1 regulation, which was significant during aging but not significant post-blood meal (Fig. 4). None of the rest of the antennally expressed *Grs* demonstrated this pattern of regulation (Fig. S5A).

#### Ionotropic receptors

Reliable expression was detected for 31 of the 52 annotated *Irs* (L5 gene set). Twenty-seven *Ir*s were age- and state-dependently regulated following a similar pattern of abundance to that of the *Or*s (Fig. 4 a left, motif 1). The sum of the abundance of the three Ir co-receptors (*Irco*s), *Ir25a*, *Ir76b* and *Ir8a*, approximated the sum of the abundance of all of the unique *Ir*s combined (Fig. 4 a; Fig. S5B; Dataset S1). The *Ircos* demonstrated an abundance ranking of *Ir25a* > *Ir8a* > *Ir76b* for 5, 9 and 10 dpe and *Ir25a* > *Ir76b* > *Ir8a* for nbf 6–8 dpe and bf 6–9 dpe female antenna (Fig. 4a left). Post-oviposition, the co-receptor *Ir76b* and the unique *Ir*, *Ir31a*, demonstrated up- and down-regulation compared to nbf females 10 dpe, respectively (Fig. S5B right).

#### Non-canonical chemoreceptor-related families

Fourteen of the 15 annotated *Trps* were reliably detected in the antennae of, at least, one of the ages under investigation (Fig. S6A). Six *Trps* from the canonical Trp (*Trp* and *TrpL*)*,* TrpA (*painless_1, painless_2*, *waterwitch_1, waterwitch_2*) and TrpM (*TrpM*) subfamilies, demonstrated age- and state-dependent regulation with *painless_2*, *waterwitch_1* and *TrpM* following the motif 1 pattern of abundance (Fig. 4 a left) and *Trp, TrpL* and *waterwitch_2*, following a motif 2 pattern of abundance (Fig. 4b left). While *TrpM* was both age- and state-dependently regulated (Fig. 4 a), *TrpA1*, was not regulated in the antenna of either nbf or bf females (Fig. S6A).

Seven of the 30 annotated *pickpocket* genes were reliably detected in the antennae of nbf and bf females (Fig. S6B). Two *Ppks* demonstrated age-dependent regulation (Fig. S6B left). The pattern of abundance of *Ppk14228* recapitulated motif 1 in age regulation only, while the increase in abundance in *Ppk02575* was observed 24 h later, between 6 and 7 dpe (Fig. S6B left). None of the *Ppks* exhibited differential abundance between the antenna of nbf and bf females (Fig. S6B right).

Both genes encoding for the sensory neuron membrane proteins, Snmp 1 and 2, were among the ten of 15 annotated *SCRB*s, that were reliably detected in the antennae of nbf and bf females (Fig. S6C). The pattern of abundance over time and across states for five of these *SCRB*s, including *Snmp1*, reflects motif 1 (Fig. 4 a), whereas two of the other regulated *SCRB*s demonstrated motif 2 (Fig. 4 b). *Snmp2* revealed a different age- and state-dependent pattern with a gradual increase in abundance from 5 to 8 dpe followed by a significant decrease in abundance by 9 dpe in nbf female antennae (Fig. S6C left), and in bf female antennae, the abundance increased significantly 24 h pbm (Fig. S6C middle).

#### Soluble odorant binding proteins

Odorant binding protein (*Obp*) and chemosensory protein (*Csp*) genes were highly abundant in the antennae of both host-seeking and blood fed 5–10 dpe female *Ae. aegypti* (Dataset S1). Thirteen *Obp*s were among the 30 most abundant genes in the female antennae (Dataset S1). Thirty-five of the 52 annotated *Obp*s were reliably detected, 14 of which were both significantly age- and state-dependently regulated, while three others were only age-dependently regulated (Fig. S7A left) and another three were only state-dependently regulated (Fig. S7A middle). The predominant pattern of expression over time and across states followed motif 1 (Fig. 4 a), while six *Obp*s followed a motif 2 pattern of abundance (Fig. 4 b). Post-blood meal, two new patterns emerged for the motif 2 (Fig. 4 b; motif 2 bf’ and bf”). The first was similar to motif 2, but in which 72 h pbm did not differ from 48 h pbm (*Obp18*, *Obp19*, *Obp21*; Fig. 4 b middle; motif 2 bf’), and the second was entirely different, decreasing in abundance from 0 to 48 h pbm and then increasing gradually until 120 h pbm (*Obp25*, *Obp57*, *Obp64*; Fig. 4 b right; motif 2 bf”). The *Csp* family in *Ae. aegypti* includes 17 *Csps*, eight of which were reliably detected in the female antennae, with four age- and four state-dependently regulated, respectively (Fig. S7B).

#### Putative odorant degrading enzymes

Two of the 95 reliably detected *Ae. aegypti* cytochrome P450 monooxygenases (*CYP*s; 130 annotated) were found among the top 50 most abundant genes in the antennae (Dataset S1). Of the 48 and 50 *CYP* genes exhibiting age- and state-dependent regulation, respectively (Fig. S8), 10 *CYP*s demonstrated changes according to motif 1, while 16 *CYP*s exhibited changes according to motif 2 (Fig. 4). Six *CYPs* were found to be differentially abundant in the antennae of 10 dpe females following oviposition compared with nbf females, with five more abundant post-oviposition and one more abundant in nbf female antennae (Fig. S8 right). Seven of the eight annotated glutathione-S-transferases (*GST*s) demonstrated reliable expression, with the three most abundant *GST*s being 40 to 200 times more abundant than the four *GST*s that exhibited abundances less than 7 TPM. These four low abundant *GST*s showed differential age- and state-regulation, with one demonstrating higher abundance in the antennae of nbf females compared to those post-oviposition (Fig. S9A). The three UDP-glucosyltransferases (*UDP-GST*s) exhibited a similar abundance to the low abundance *GST*s (4–7 TPM), with only the most abundant exhibiting motif 1-like age- and state-dependent regulation (Fig. S9B). Six of the 17 annotated carboxyl/cholinesterases (*CCE*s) were reliably detected in the antennae of nbf and bf females, with the two regulated genes demonstrating motif 1 in abundance both age- and state-dependently (Fig. S9C).

### Regulation of modulatory genes

#### Neuropeptide signalling

Genes encoding for neuropeptides and their receptors involved in the modulation of olfaction in insects, particularly in relation to mating, feeding and reproductive status (see discussion), were expressed in the antennae of nbf and bf 5–10 dpe female *Ae. aegypti*, and were regulated with age and feeding status (Fig. S10AB). The transcripts of the neuropeptides insulin-like peptide (ILP), short neuropeptide F (sNPF) and tachykinin (TK) followed a similar pattern of abundance in the antennae of nbf and bf females from 5 to 10 dpe (Fig. S10AB; motif 2). In contrast to the neuropeptides, their cognate receptors generally displayed regulation according to motif 1 (Fig. S10AB). The *insulin receptor* (*InR*) peaked in abundance 24 h pbm and gradually returned to low abundance (ca. 1 TPM) at 120 h pbm. The abundance of the TK receptor (*GPRTAK2*) demonstrated a longer period (ca. 72 h) oscillation to that of the neuropeptides, and the *sNPF receptor* (*GPRNPY7*) abundance increased to a plateau at 7–8 dpe and then decreased to below reliable expression at 9 and 10 dpe (Fig. S10AB). The reduction in neuropeptide transcript abundance demonstrated for the antennae of nbf 5–6 dpe females is reflected 0–24 h post-blood meal, with a subsequent oscillation (48 h period) in abundance in the antennae of bf females terminating in a significant increase in abundance at 120 h pbm (Fig. S10AB). The *GPRTAK2* abundance profile mirrored that of *TK*, while *GPRNPY7* demonstrated a counter oscillation abundance profile to that of *sNPF*, albeit at levels below 1 TMP. The abundance of *InR* increased from 0 h pbm, peaked at 72 h pbm and subsequently decreased to near threshold levels at 120 h pbm (Fig. S10AB). The majority of the reliably detected neuropeptides demonstrated motif 2, while the response profiles of the receptors were more variable, demonstrating both motif 1 and 2, among others (Fig. S10AC). In total, 14 pairs of peptides and receptors were reliably detected together in the antennae. In addition, genes encoding 12 peptides and 15 receptors were expressed without expressing their cognate receptor or peptide genes, respectively, indicating that there are additional intrinsic and extrinsic regulatory pathways operating in the antennae of female *Ae. aegypti* during the first gonotrophic cycle (Fig. S10AC).

#### Biogenic amine signalling pathways

In the antennae, the transcript abundance of the enzymes involved in generating biogenic amines were differentially regulated post-blood meal (Fig. S11A). In the dopamine synthesis pathway, *tyrosine hydroxylase* (*TH*) abundance demonstrated motif 1-like age- and state-regulation, with an increased abundance post-blood meal that resulted in a significant difference at 96 h pbm. Two dopamine receptors (*Dop1R1*, *DOP2*) were reliably detected in the antennae, with *Dop1R1* increasing in abundance until 6 dpe, and with a higher level of abundance of *DOP2* from 48 h pbm to 96 h pbm (Fig. S11A). The abundance of the dopamine transporter, DAT, reflects that of the enzymes and receptors, but with the pattern exaggerated, resulting in more significant differences (Fig. S11B). In the serotonin and octopamine synthesis pathways, the antennal abundance of *tryptophan hydroxylase* (*TPH*), as well as of *tyrosine decarboxylases* (*Tdc1*, *Tdc2*) and *tyramine beta hydroxylase* (*TßH*), showed motif 2-like regulation, demonstrating a tri-phasic response (Fig. S11A). The designated serotonin transporter, *SerT*, also demonstrated this abundance profile, along with only one of the other eight paralogues designated in the *Ae. aegypti* genome (Fig. S11B). While none of the three octopamine receptor, nor the tyramine receptor, transcripts were reliably detected in the antennae, three of the five serotonin receptor transcripts were. Two serotonin receptor transcripts, the *5-HT7R*s (AAEL025125, AAEL027242), demonstrated a motif 1-like regulation of abundance, which is in counterpoint to the regulation of *TPH* in the antennae, while the other serotonin receptor transcript (*5-HT2BR*, AAEL019805) was not regulated (Fig. S11A).

#### Neurotransmitter signalling

Antennal transcript abundance for acetylcholine biosynthetic enzyme, *ChAT*, vesicular transporter, *VAChT*, degrading enzyme, *AChE*, and reuptake transporter, *ChT*, followed a motif 1 age- and state-dependent regulation (Fig. S12). Three of the nine annotated nicotinic acetylcholine receptors (*AChR1a*, *AChR2a* and *AChR2b*) demonstrated reliable expression throughout the first gonotrophic cycle, with *AChR1a* decreasing in abundance until 6 dpe, and increasing in abundance post-oviposition (Fig. S12). *AChR2a* and *AChR2b* followed a motif 2-like age- and state-dependant regulation (Fig. S12). It is interesting to note that the pattern of abundance described by cholinergic signalling in the antennae reflects that of the majority of the chemosensory receptor transcripts. Transcripts of six glutamate receptors were found to be reliably detected in the antenna, with only two ionotropic receptors, *clumsy2* and *GluR*_*A/B*_, exhibiting a motif 2-like age- and state-dependant regulation (Fig. S13).

Three of the four reliably detected GABA receptors demonstrated a motif 1 age- and state-dependant regulation, while the fourth receptor demonstrated a motif 2-like regulation (Fig. S13). In the antennae of *Ae. aegypti*, all members of the GABA signalling pathway, biosynthetic enzymes (*Gad1–4*), as well as vesicular and reuptake transporters, were reliably detected during the first gonotrophic cycle (Fig. S13). All of these transcripts demonstrated a motif 2-like age- and state-dependant regulation, with the exception of Gad4, which was not regulated (Fig. S13).

### Regulatory genes

A screen to identify potential transcription factor binding sites in the region 1 kb 5′ to the start site of all of the reliably detected genes from each gene family under consideration revealed that genes demonstrating motif 1- and motif 2-like abundance profiles displayed differential enrichment of transcription factor binding sites. Genes demonstrating motif 1-like profiles were significantly over-represented in the binding sites from the transcription factors *Chromatin-linked adaptor for MSL proteins* (*Clamp*), *odd paired* (*opa*), *Signal-transducer and activator of transcription protein at 92E* (*Stat92E*), *suppressor of Hairy wing* (*su (Hw)*) and *tailless* (*tll*), and significantly under-represented in the sites from *CTCF* (F test, *p* < 0.05). Of these, only *Clamp*, *Stat92E* and *su (Hw)* were reliably expressed, and none of these displayed age- or state-dependent differential abundance (Fig. S14). Those genes with motif 2-like profiles revealed an over-representation of binding sites for the transcription factors, *Big brother::runt* (*Bgb::run)*, *doublesex-Mab related 99B* (*dmrt99B*), *pleiohomeotic* (*pho*) and *tramtrack* (*ttk*), while *onecut*, *nubbin* (*nub*), *dorsal* (*dl*) and *slow border cells* (*slbo*) were significantly under-represented compared to the overall gene set (F test, *p* < 0.05). Of these, *pho*, *ttk*, *nub* and *dl* displayed motif 1-like regulation, while *Bgb*, *run* and *onecut* were not age- or state-dependently regulated, and *slbo* and *dmrt99B* were not reliably expressed (Fig. S14).

Of the eight previously identified transcription factors as being involved in *Or* regulation, *onecut*, *abnormal chemosensory jump 6* (*acj6*), *fruitless (fru*), *Ecdysone-induced protein 93F* (*E93*), *single-minded (sim*), *48 related 1* (*Fer1*), *Pmd3* and *Xeroderma pigmentosum D* (*Xpd1*) [30–32], only three were age- and state-dependently regulated, with *acj6* and *fru* demonstrating a motif 1-like change in abundance and *E93* demonstrating higher abundance between 48 and 96 h pbm (Fig. S14). The other transcription factors identified in this study revealed either a motif 1-like regulation, or were not regulated with age or state (Fig. S14).

## Discussion

The current study presents an in-depth analysis of the molecular apparatus involved in the regulation of the gene expression in the antennae of female *Ae. aegypti* throughout the first gonotrophic cycle, with the aim to improve our understanding of the dynamic nature of the peripheral olfactory system. Antennal transcription in host-seeking females is regulated as the mosquito ages [this study, 17], reflecting the dynamic nature of odour-driven behaviour of blood-seeking females during the first gonotrophic cycle [9, 10, 17], and demonstrating the importance of using age-matched cohorts to identify genes regulated post-blood meal. The age- and state-dependent abundance profiles of chemosensory and neuromodulatory genes in the antennae appear to be regulated through at least two concerted regulatory pathways, with links to a series of transcriptional factors, providing evidence that the peripheral olfactory system of a female mosquito is regulated by both the synchronised expression of subsets of genes, as well as the unique expression patterns of individual genes.

As the *Ae. aegypti* mosquito ages, the female generally becomes competent to host seek between 3 and 5 dpe, with the majority of individuals subsequently engaging in this activity [8, 9]. The proportion of females engaging in host seeking, however, appears to vary with age, increasing overall until 9–11 dpe, while exhibiting diel and inter-diel oscillations (Fig. 1) [8, 9]. By controlling for the age of the female and the time of day of the antennal dissections, this study aimed to reduce the variation in transcript abundance attributable to diel and circadian rhythms [26, 27], and thus uncover any variation ascribable to age and gonotrophic state. The demonstrated age-dependent change in the abundance of the clock gene transcripts reflects the crepuscular activity demonstrated by host-seeking females as they age between 5 and 10 dpe [33], indicating the effective control of the diel variation in this study.

### Overall antennal gene expression

The dynamic GO profiles in the antennae of nbf females 1, 3 and 5 dpe [17], and those spanning 5–10 dpe [this study], support the proposed two age-dependent processes occurring during the first 10 dpe: 1) a maturation of the peripheral olfactory system, which is completed by 5 dpe, and 2) an ongoing systemic modulation of the peripheral olfactory system of mature host-seeking females, which continues until at least 9 dpe. The major molecular functional classes identified as changing with age in the antennae of *Ae. aegypti* females have also been shown to change in the heads of *Drosophila melanogaster* [34] and the heads and thoraces of *Anopheles gambiae* [35], as the insects age. As host-seeking females age from 5 to 10 dpe, overall gene expression appears to oscillate between two states:1) that found at 5 dpe, and again at 9 and 10 dpe, and 2) that found at 6–8 dpe. The gene expression observed from 1 to 5 dpe in *Ae. aegypti* reflects previous behavioural results describing an increasing propensity to host seek, which oscillates as it increases, in *Ae. aegypti* and the African malaria mosquito, *An. gambiae* [8, 10, 16, 17], as well as a similar increasing oscillating propensity to blood feed over the same time period in *Ae. aegypti* [9]. Moreover, periodicity is evident in both behaviours extending past adult maturation, through to 9–11 dpe [9, 16]. Thus, during the first two weeks of the adult life of the female mosquito, the modulation of behaviours that rely heavily on the detection and transduction of chemosensory cues is reflected in the differential regulation of genes with various molecular functions related to chemosensation. Our results suggest that the modulation of the peripheral olfactory system of an aging adult host-seeking female *Ae. aegypti* is largely a result of selective protein synthesis, protein and odorant binding, and protein and odorant degradation.

While increasing age at the time of the blood meal has been shown to shorten and reduce the intensity of the post-blood meal host odour refractory period [19], young female mosquitoes (ca. 5 dpe) become refractory to host odours within 12–24 h pbm [19] returning to host seeking within 24 h after oviposition [11]. The majority of genes that changed abundance in the antennae post-blood meal, compared to nbf females of the same age, occurred during the host-seeking refractory period, peaking 12–24 h prior to oviposition, with approximately equal numbers being up- and down-regulated [this study, 21]. It is also only during this refractory period that there is a down-regulation in the gene abundance underlying the translational machinery [36, this study]. While all the differentially abundant structural constituents of ribosome encoding genes exhibit significantly lower abundance in the antennae, as all but two do in the overall body, the peak time and intensity of down-regulation differ (whole body: 54 genes 48 h pbm vs. antennae: 101 genes 96 h pbm), suggesting that the translational machinery in the antennae is differentially regulated. Taking into account the large number of genes displaying up- and down-regulation at 96 h pbm, together with the down-regulation of more than two thirds of the translational machinery in the antennae, and the delay between transcription and translation, it is possible that the peripheral olfactory system at 96 h pbm is preparing for the transition from oviposition to host seeking within the next 24 h. This is supported by the significantly fewer differentially abundant genes identified between the antennae of nbf and bf females at 10 dpe compared with 9 dpe.

### Chemosensory gene expression

#### Age-dependence

The age-related changes in chemosensory gene abundance in nbf females are reminiscent of the previously demonstrated varied proportions of *Ae. aegypti* females engaging in host seeking and blood feeding over the first 10 dpe (Fig. 1) [9, 10, 17], which describe apparent oscillations in behaviour up to at least 11 dpe [9, 10]. The majority of reliably expressed *Ors*, *Irs*, *SCRBs*, *Obps* and putative *Odes* age-dependently oscillate between two relative abundance levels, those at 5, 9 and 10 dpe, and those at 6–8 dpe. These differences can be described by either of two motifs. Motif 1 describes a lower abundance levels in antennae at 5, 9 and 10 dpe than those at 6–8 dpe in host-seeking females, and a significant increase in abundance 96 h pbm, while motif 2 describes an inverse pattern of abundance. As nbf females age, host-seeking behaviours change in intensity and frequency, with females becoming more avid, taking more risks and increasing the frequency of multiple blood meals in order to achieve a sufficient blood meal to complete egg development [11, 37]. In addition, the proportion of females taking nectar meals may reduce as the mosquito ages [6]. Almost one-fifth of the reliably expressed *Ors*, *Irs*, *SCRBs*, *Obps* and putative *Odes* exhibit differential abundance in the antenna between 5 and 10 dpe host-seeking females, which may indicate a role for these olfactory-related genes in these age-dependent behavioural changes.

#### Abundance and function

The functional significance of the differences in chemosensory gene abundance is yet unclear. Previous studies have implied a link between chemosensory transcript abundance and olfactory sensory neuron (OSN) sensitivity [23, 25, 38]. The differential age- and state-dependent changes in OSN sensitivity to their specific volatile organic compound ligands appears to correlate with the differential abundance of the cognate chemoreceptors in the antennae [38] and maxillary palps of female mosquitoes [23, 25]. As such, the post-blood meal changes in OSN sensitivity described by Siju et al. [39] and Chen et al. [40] may also be linked to changes in the abundance of select chemosensory-related genes, although a comprehensive functional analysis of *Ae. aegypti* Ors, Irs, Grs and Obps is currently lacking.

Among the few antennally expressed *Ors* in adult *Ae. aegypti* that have been functionally characterised, Or2 (indole-sensitive) [41, 42] was neither regulated with age nor blood meal status, while Or4 (sulcatone-sensitive) [43], Or10 (indole-sensitive) [41, 44], Or14 (weakly 4-methylphenol-sensitive) [45] and Or15 (phenethyl propionate-sensitive) [45], all increased significantly in abundance from 5 to 6 dpe in the antennae of host-seeking females. The majority of the volatile ligands for these Ors have been identified as candidate attractants for host-seeking *Ae. aegypti* [46]. Of these functionally characterised Ors, only *Or14* was found to be regulated post-blood meal, during the time of pre-oviposition behaviour, similar to the 4-methylphenol-sensitive neuron, sbtII3A, in the antennae [39].

The functionally characterised Irs that followed the motif 1 pattern of differential abundance were the homologues of the hygroreceptive *D. melanogaster Ir40a* [47], the heat-sensitive *An. gambiae Ir21a* [47], the short-chain carboxylic acid-sensitive *An. gambiae Ir75k* [48], and the polyamine-sensitive *An. gambiae Ir41a* [48]. Olfactory sensory neurons with sensitivity to short-chain carboxylic acids [39] and polyamines [40] showed increased sensitivity to their ligands shortly before oviposition. Thus, the demonstrated increase in abundance of select *Irs*, and the sensory neurons tuned to their cognate ligands, coincide with the onset of oviposition site selection (96 h pbm) [49–53].

Only two Obps have been functionally characterised in *Ae. aegypti*, Obp22 and Obp39, which are sensitive to long chain fatty acids and the mosquito oviposition pheromone (5 *R*,6 *S*)-6-acetoxy-5-hexadecanolide, respectively [54, 55]. While *Obp39* was not age- or state-dependently regulated, *Obp22* demonstrated motif 2-type regulation. This suggests that the sensitivity to long chain fatty acids oscillates with age and is more likely to play a role in host-seeking females of the same age. The lack of functional data for Obps has been augmented by structure-function modelling [56]. Of the modelled *Obp*s, the 75% that demonstrated differential abundances following motif 1 predicted permethrin as the primary or secondary key ligand, whereas 100% of those demonstrating a motif 2-like differential abundance ranked ammonia within the top three predicted ligands [57]. The differential age- and state-dependent regulation of *Obp*s suggest different roles for these Obps in the antennae of *Ae. aegypti*. Given the paucity of functional data for the chemosensory receptors and proteins of *Ae. aegypti*, the ecological and functional significance of the observed differential abundance requires further investigation.

#### Abundance and behaviour

In the absence of functional data, age- and state-dependent patterns of gene abundance in the antennae have the potential to provide insight into the regulation of host-seeking, and pre- and post-oviposition odour-mediated behaviours [20–25, 58]. The clearest trend in patterns of gene abundance described during the first gonotrophic cycle was that post-blood meal regulation occurred in the majority of chemosensory genes just a few hours prior oviposition (this study, [21]), when females are exhibiting oviposition site seeking [13, 14]. Furthermore, the overall patterns of abundance demonstrated for the chemosensory gene families in response to maturation [17], aging and reproductive state [this study], indicate a concerted regulation of the peripheral olfactory system, which coincides with the changing intensity of odour-mediated behaviours through time [25, 38] and with physiological state [20, 23].

Many chemosensory genes were not significantly regulated by age or state, while some demonstrated a different pattern of regulation compared to motifs 1 and 2. One example of a set of chemosensory genes that did not follow motif 1- or 2-type regulation was the pattern exhibited by *Or117*, *Obp22* and *Obp64*. These genes were the only chemosensory genes to be down-regulated during maturation [17], and are among only 16 genes that were up-regulated within 24 h of a blood meal, returning post-oviposition to abundance levels in the antennae that did not differ from nbf females. Results obtained in this and a previous study [17] imply that these genes are involved in the regulation of host seeking in *Ae. aegypti*, as their abundance level inversely coincides with the propensity of females to respond to host odour [11, 19–23]. Post-oviposition, the host-odour refractory period subsides within 12–24 h [19] and host-seeking females demonstrate a higher proportion of females engaging in host seeking than females of the same age which have not previously experienced a blood meal [11, 19]. This increase in host seeking post-oviposition corresponds with the differential abundance of 13 chemosensory-related genes in the antennae of nbf and bf females 10 dpe, seven of which were putative Odes, suggesting that the increased host-seeking activity may be related, at least in part, to a change in the efficiency of the clearance of salient odorants from the antennae.

### Non-canonical chemoreceptor-related families

Transient receptor potential receptors (*Trps*) and *Ppks* are gene families encoding polymodal receptors sensing a wide variety of stimuli, including mechanotransduction, proprioception, neurotransmission, and fluid and electrolyte homeostasis. (e.g. [59, 60]). The *Trps* detected in this study are from six of the seven defined *Trp* subfamilies [59]. In other insects, *Trp* and *TrpL* are implicated in vision and chordotonal mechanosensory amplification, and for *TrpL* also in sensing cold [59]. The differential age-dependent regulation of the *waterwitch* paralogues suggests different functional roles for the paralogues of these *TrpAs*. *waterwitch* plays a role in sensing moist air, as well as mechanosensory signal amplification in the Johnston’s organ, particularly in wind- and gravity-sensing, together with *painless* [59]. In *Drosophila*, both *TrpM* and *TrpA1* have been implicated in chemosensation, sensing modified terpenes, such as menthol [61]. The *Ppks* described in this study, expanding on those described by Matthews et al. [21], were not homologous to any of the chemosensitive *Ppks* described in other insects [60]. The *D. melanogaster* homologues of the two age dependently regulated *Ppks* in this study, *ppk*, *ppk22*, *ppk24*, *ppk26* and *ripped pocket*, and have been implicated in mechanosensation [62, 63]. Chemosensory, thermal and humidity stimuli are known to regulate both host and oviposition behaviours in mosquitoes [50, 52, 64], but how these sensory cues interact to generate these behaviours remains opaque. The differential regulation of the various *Trps* and *Ppks* reflects this complexity, and highlights the need for further functional characterisation and investigation into multi-modal integration.

### Mechanisms for gene regulation

#### Neuromodulation

Neuropeptides and their receptors are involved in the modulation of olfaction in insects, particularly in relation to mating, feeding and reproductive status [65]. In *D. melanogaster*, insulin-like peptide (ILP), short neuropeptide F (sNPF) and tachykinin (TK) signalling modulate the sensitivity of OSNs in response to feeding state, both intrinsically and extrinsically [66–68]. The contrast between the similarity in regulation of the neuropeptides and the differences among the receptors reflects the previously demonstrated role of neuropeptide signalling in OSNs. The release of ILP, sNPF and TK has been shown to autoregulate OSNs and the strength of their synapses with projection neurons, affecting odorant signalling of OSN classes and odour valence (for review see [69]).

The biogenic amines are involved in the modulation of the peripheral olfactory system in insects, including mosquitoes [70–72]. In the heads of *Ae. aegypti* females, biogenic amines are regulated post-blood meal during the first gonotrophic cycle, with the levels of serotonin and dopamine, reduced and increased, respectively, within 5 min post-blood meal, and then maintained at these levels until at least 72 h pbm [72]. The levels of octopamine in the heads post-blood meal change over three time scales, fast, intermediate and slow, with a marked reduction in octopamine observed during the phase of inactivity induced post-blood feeding [72]. The transcript abundance of the enzymes involved in these biogenic amine synthesis pathways were differentially regulated in the antennae post-blood meal, in a tri-phasic manner, similar to that described for the biogenic amines in the head [72], while the two serotonin receptor transcripts were regulated in counterpoint to this regulation. This pattern of abundances suggests a similar mechanism for the regulation of biogenic amine release as described above for the neuropeptides.

Afferent signalling associated with insect antennae has been largely described as cholinergic (e.g. [73]). While insects have the ability to synthesise choline from serine, the bulk of choline involved in neuronal signalling and phospholipid membranes is from dietary sources, and is assiduously recycled by neurons [74]. As of yet, no afferent glutamate signalling has been described in the insect peripheral olfactory system, which is supported in *Ae. aegypti*, as none of the vesicular (*VGluT*) or neuronal reuptake transporters (*EAAT3*) were reliably detected in the antennae of females during the first gonotrophic cycle. Synapses between OSNs and excitatory glutaminergic lateral neurons, however, have been described in *D. melanogaster* [75], indicating a need for glutamate receptors to be expressed in OSNs. Moreover, GABAergic signalling has been well-described at synapses between lateral inhibitory neurons in the antennal lobe and OSNs [76, 77], indicating a need for GABA receptors in OSNs. However, while GABA signalling in antennal OSNs of locusts and moths has been postulated [78, 79], it has not yet been shown in mosquitoes. The reliable detection of all of the components of the GABA signalling pathway is a strong indication that there is significant GABA signalling associated with cells originating within the antennae of female *Ae. aegypti*, and it would be interesting to investigate whether this is associated with OSNs.

#### Transcription factors

A discussion of the regulation of antennal gene expression through the first gonotrophic cycle should include the transcription factors, a set of motif-specific DNA-binding proteins directly involved in the qualitative and quantitative regulation of gene transcription. While the transcription factors with binding sites enriched up-stream of genes demonstrating a motif 1-type regulation were not age- or state-dependently regulated, more than half of those with binding sites enriched up-stream of genes demonstrating a motif 2-type regulation displayed a motif 1 pattern of regulation. In previous studies, cis-regulatory elements (CREs) were identified for 31 transcriptional factors in *Ae. aegypti*, of which eight transcription factors were found 1 kb 5′ to *Ors* in *Ae. aegypti*: *onecut*, *acj6*, *fru*, *E93*, *sim*, *Fer1*, *Pmd3* and *Xpd1* [30–32]. In this study, 22 of the 31 previously identified transcriptional factors [31] were reliably expressed in the antennae, and 16 of these demonstrated enriched binding sites 1 kb 5′ to chemosensory-related genes. Only *onecut* had transcription factor binding sites that were enriched up-stream of *Ors*, but none of the other chemosensory gene families, suggesting a role in regulating Or-driven behaviours, such as host seeking. Another transcriptional factor, *fru*, has recently been implicated in the regulation of behavioural sensitivity to host odour following CRISPR-Cas9 knock-out in *Ae. aegypti* [Basrur et al., *preprint* 10.1101/2020.09.04.282434]. The demonstrated pattern of transcription factor regulation provides a tentative substrate for the observed modulation of chemosensory-related gene abundance, and provides evidence that these gene sets may be under differential, concerted regulation in the antennae throughout the first gonotrophic cycle.

## Conclusions

Here, we have demonstrated that the peripheral olfactory system in age-matched host-seeking and blood-fed female *Ae. aegypti* is regulated throughout the first gonotrophic cycle. We provide clear evidence that the age of the female affects antennal transcription differentially, rather than globally. This is notable, as this highlights the dynamic regulation of genes associated with olfactory-driven behaviour over time in host-seeking females. By controlling for age, we identify a larger number of transcripts regulated post-blood meal than previously reported. In addition, we find that the chemosensory-related gene families appear to be age- and state-regulated through at least two concerted regulatory pathways, linked to subsets of transcriptional factors. This survey provides a substrate for future studies towards the development of novel vector control targets.

## Methods

### Rearing and tissue collection

The laboratory culture of *Aedes aegypti* (Rockefeller strain) used in this study has been reared continuously in our facilities since 2005 (founding eggs courtesy of Professor Ring Cardé). From the same cohort of *Ae. aegypti*, two populations of adult mosquitoes were reared separately under standard laboratory culture conditions (27 ± 2 °C; 70 ± 2% relative humidity; 12 h:12 h light-dark photoperiod). One population was blood fed 5 days post-emergence, while all mosquitoes were provided with ad libitum access to 10% sucrose solution. Over the subsequent 5 days, the antennae of the adult females from both populations, were dissected progressively at 0–1 h, 24 ± 1 h, 48 ± 1 h, 72 ± 1 h, 96 ± 1 h, and 120 ± 1 h pbm. All dissections were made between zeitgeber time (ZT) 5 and ZT 7, i.e. the peak activity period for adult *Ae. aegypti* females [33]. Prior to oviposition, females were segregated individually in tall Petri dishes (diameter 12 cm, height 6 cm; Semadeni, Ostermundigen, Switzerland), and at 72 h pbm provided with an oviposition substrate, i.e. a cone-shaped filter paper. Only females that oviposited within the next 24 h were included in the 120 h pbm dissections. Female mosquitoes were anaesthetised on ice before dissection. The tissue was collected into RNAlater® (Thermo Fisher Scientific, Gothenburg, Sweden), and stored at 8 °C overnight and then transferred to − 80 °C until RNA extraction. Three biological replicates of ca. 150 pairs of antennae each were collected per time point and state, non-blood fed (nbf) and blood fed (bf).

### RNA extraction and sequencing

Tissues were homogenised using a Vibra-Cell sonicator (V*CX-*130, Sonics and Materials, Newtown, CT, USA) for 10 cycles at 70% amplitude, 1 s on and off pulses, repeated three times, interspersed with 30 s incubations on ice. Total RNA was extracted using an RNeasy Mini Kit (Qiagen, Sollentuna, Sweden) according to the manufacturer’s protocol, including the on-column RNase-free DNase I treatment (Qiagen). The RNA was quantified fluorometrically (Qubit, Life Technologies, Stockholm, Sweden) and then stored at − 80 °C prior to shipment on dry ice to Beijing Genomics Institute (BGI, Hong Kong, China) for library construction and paired-end Illumina sequencing (TruSeq) of the cDNA libraries (Illumina HiSeq 4000).

### Read mapping and gene annotations

Prior to mapping, adapter sequences were removed from the raw reads and low-quality bases from the start and end of each single read were clipped in a sliding window approach, according to BGI standard. Reads shorter than a length threshold of 40 nt were also removed. CLC Genomics Workbench 11.0 (http://www.clcbio.com; Qiagen, Vedbæk, DK) was used to map the cleaned sequenced reads to the reference genome (VectorBase, *Aedes aegypti* LVP_AGWG AaegL5 genome with gene set AaegL5.1 [80]). On average, 90% of all reads from each library mapped onto the genome.

### RNA-Seq and differential expression analyses

RNA-Seq analyses were performed using CLC Genomics Workbench 11.0.1. Transcripts per million (TPM) was chosen to quantify transcript abundance, to enable comparison with Matthews et al. [21]. A threshold of less than 1 TPM maximum counts across all libraries was used as a cut off to determine reliably detected genes. Differential transcript abundance was determined using the negative *ß*-binomial general linear model algorithms in CLC Genomics Workbench 11.0.1, which are similar to those used by edgeR and DESeq [81]. To control for false discovery rate (FDR), the Benjamini-Hochberg correction was applied [82]. This analysis generated fold changes (FC) and FDR corrected *p*-values that were used to detect differential expression. Genes that exhibited a FC > 2 and FDR *P* < 0.05 were considered to be significantly differentially abundant.

### Sequencing statistics

Paired-end Illumina sequencing of antennal mRNA from 5 to 10 days post-emergence non-blood fed (nbf) and age-matched blood fed (bf) cohorts, with three biological replicates each, generated a total of 36 libraries spanning the adult female’s first gonotrophic cycle. A combined sequencing depth of over 804 million cleaned reads, i.e. on average 22 million reads per library (Dataset S1), was obtained from these FASTQ libraries. The reliable expression (> 1 TPM) of 442 out of the 450 total annotated core eukaryotic genes (CEGs), a set of housekeeping genes ubiquitously conserved across eukaryotes [83], in each of the six time points from the antennal libraries (Dataset S2), describes good sequence coverage within each of the transcriptomes. Additionally, a comparison of the CEGs between the nbf and bf cohorts of the same age at each of the six time points revealed that < 4.5% of the genes exhibited a significant (False Discovery Rate p-value; FDR *P* < 0.05) > 2-fold difference in abundance (Fig. S15; Dataset S2). This suggests that these criteria represent a rational cut-off for the level of significance for the subsequent screens. Comparisons among the replicates of each time point and feeding state furthermore revealed a maximum of 4 CEGs exhibiting a > 2-fold change, with 25 of the 36 libraries demonstrating 0–1 genes exhibiting a > 2-fold change (Dataset S2; Fig. S16).

### Transcription factor binding site analyses

Transcription factor binding sites were identified using CiiiDER v. 0.9 [84]. A FASTA file including all of the nucleotide sequences 1 kb 5′ to the start site of each gene investigated was created using Biomart (VectorBase.org). From this file, two files were generated containing the sequences upstream of motif 1 and motif 2. Two other files were made from the remaining sequences. These four files were used as input and enhancement references to identify the transcription factor binding sites, using the *Drosophila* non-redundant transcription factor binding site database (Jasper core non-redundant Dm; http://jaspar.genereg.net/html/DOWNLOAD/JASPAR_CORE/pfm/), found differentially upstream of genes demonstrating the transcript abundance profiles motif 1 and motif 2.

## Supplementary Information

**Additional file 1: Figure S1**. Abundance of the clock gene transcripts. Transcript abundances of *period*, *clock*, *timeless*, *vrille*, *cycle* and *Pdp1* in the antenna of non-blood fed (black solid line) and blood fed (grey solid line) *Aedes aegypti* throughout the first gonotrophic cycle (5 to 10 days post-emergence; dpe). The error bars represent the standard error of the mean.

**Additional file 2: Figure S2**. Reliable expression of antennal genes by molecular function. Proportions of reliably detected genes in the antennae of 5 to 10 days post-emergence (dpe) host-seeking adult female *Aedes aegypti* classified by a level 3 molecular function gene ontology (**A-F**). Inset: Total number of reliably detected genes from each age group can be determined by the sum of those with GO annotation (white) and those without (green).

**Additional file 3: Figure S3**. Comparison of odorant receptor abundance between this study and Matthews et al. (2016) [21]. The sugar fed condition in our data set is calculated as the average of all ages and replicates of nbf. The “bf” is calculated as the average of all bf replicates of 24 and 48 h pbm. The “ovi” is calculated as the average of all of the replicates from 48, 72 and 96 h pbm. These decisions were made based on the similarities with the described protocol in Matthews et al. 2016 [21]. Please note that there were 36 previously identified odorant receptor (*Or*) genes that are no longer included, or have been collapsed into variants of other *Or*s, in the present L5 annotation. Additionally, *Or20* and *Or115* are represented in the current L5 annotation by two genes each.

**Additional file 4: Figure S4**. Odorant receptor transcript abundance is age- and state-dependent. Odorant receptor transcript abundance in 5 to 10 days post-emergence (dpe) non-blood fed (nbf; green; left) and age-matched blood fed (bf; brown; middle) *Aedes aegypti* female antennae. Comparisons between nbf (black) and age-matched bf (blue) antennal transcript abundance are described by fold change (right). Permanent gene identifiers along with the common gene names are to the left. Ball and stick diagrams represent the general trend in abundance demonstrated by this gene family (motif 1; bottom right). Asterisks between two age groups denote significant difference (> 2-fold change; FDR *P* < 0.05). Asterisks to the far right of each table indicate significant differences between 5 and 10 dpe. Above the horizontal grey line are the transcripts with an overall abundance greater than 1 TPM.

**Additional file 5: Figure S5**. Gustatory and ionotropic receptor transcript abundance is age- and state-dependent. Gustatory (**A**) and ionotropic (**B**) receptor transcript abundance in 5 to 10 days post-emergence (dpe) non-blood fed (nbf; green; left) and age-matched blood fed (bf; brown; middle) *Aedes aegypti* female antennae. Comparisons between nbf (black) and age-matched bf (blue) are described by fold change (right). Permanent gene identifiers along with the common gene names are to the left. Ball and stick diagrams represent the general trend in abundance demonstrated by these gene families (motif 1; bottom right). Asterisks between two age groups denote significant difference (> 2-fold change; FDR *P* < 0.05). Asterisks to the far right of each table indicate significant differences between 5 and 10 dpe. Above the horizontal grey line are the transcripts with an overall abundance greater than 1 TPM.

**Additional file 6: Figure S6**. Transient receptor potential, pickpocket and class B scavenger receptor transcript abundance is age- and state-dependent. Transient receptor potential (**A**), pickpocket (**B**) and class B scavenger (**C**) receptor transcript abundance in 5 to 10 days post-emergence (dpe) non-blood fed (nbf; green; left) and age-matched blood fed (bf; brown; middle) *Aedes aegypti* female antennae. Comparisons between nbf (black) and age-matched bf (blue) are described by fold change (right). Permanent gene identifiers along with the common gene names are to the left. Ball and stick diagrams represent the two general trends in abundance demonstrated by these gene families (motifs 1 and 2; bottom right). Asterisks between two age groups denote significant difference (> 2-fold change; FDR *P* < 0.05). Asterisks to the far right of each table indicate significant differences between 5 and 10 dpe. Above the horizontal grey line are the transcripts with an overall abundance greater than 1 TPM.

**Additional file 7: Figure S7**. Odorant binding protein and chemosensory protein transcript abundance is age- and state-dependent. Odorant binding protein (**A**) and chemosensory protein (**B**) transcript abundance in 5 to 10 days post-emergence (dpe) non-blood fed (nbf; green; left) and age-matched blood fed (bf; brown; middle) *Aedes aegypti* female antennae. Comparisons between nbf (black) and age-matched bf (blue) are described by fold change (right). Permanent gene identifiers along with the common gene names are to the left. Ball and stick diagrams represent the two general trends in abundance demonstrated by these gene families (motifs 1 and 2; bottom right). Asterisks between two age groups denote significant difference (> 2-fold change; FDR *P* < 0.05). Asterisks to the far right of each table indicate significant differences between 5 and 10 dpe. Above the horizontal grey line are the transcripts with an overall abundance greater than 1 TPM.

**Additional file 8: Figure S8**. Cytochrome P450 oxidase transcript abundance is age- and state-dependent. Cytochrome P450 oxidase transcript abundance in 5 to 10 days post-emergence (dpe) non-blood fed (nbf; green; left) and age-matched blood fed (bf; brown; middle) *Aedes aegypti* female antennae. Comparisons between nbf (black) and age-matched bf (blue) are described by fold change (right). Permanent gene identifiers along with the common gene names are to the left. Ball and stick diagrams represent the two general trends in abundance demonstrated by this gene family (motifs 1 and 2; bottom right). Asterisks between two age groups denote significant difference (> 2-fold change; FDR *P* < 0.05). Asterisks to the far right of each table indicate significant differences between 5 and 10 dpe. Above the horizontal grey line are the transcripts with an overall abundance greater than 1 TPM.

**Additional file 9: Figure S9**. Glutathione-S-transferase, UDP-glucosyltransferase and carboxyl/ cholinesterase transcript abundance is age- and state-dependent. Glutathione-S-transferase (GST; **A**), UDP-glucosyltransferase (UDP-GST; **B**) and carboxyl/cholinesterase (CCE; **C**) transcript abundance in 5 to 10 days post-emergence (dpe) non-blood fed (nbf; green; left) and age-matched blood fed (bf; brown; middle) *Aedes aegypti* female antennae. Comparisons between nbf (black) and age-matched bf (blue) are described by fold change (right). Permanent gene identifiers along with the common gene names are to the left. Ball and stick diagrams represent the two general trends in abundance demonstrated by these gene families (motifs 1 and 2; bottom right). Asterisks between two age groups denote significant difference (> 2-fold change; FDR *P* < 0.05). Above the horizontal grey line are the transcripts with an overall abundance greater than 1 TPM.

**Additional file 10: Figure S10**. Neuropeptide and neuropeptide receptor transcript abundance is age- and state-dependent. Paired neuropeptide (right) and neuropeptide receptor (left; **A**), transcript abundance in 5 to 10 days post-emergence (dpe) non-blood fed (nbf; green) and age-matched blood fed (bf; brown) *Aedes aegypti* female antennae. Comparisons between nbf (black) and age-matched bf (blue) are described by fold change. Permanent gene identifiers along with the common gene names are indicated. **B.** Identified modulators of insect gonotrophic behaviours, insulin-like peptide (ILP), short neuropeptide F (sNPF) and tachykinin (TK) peptides and their cognate receptors are shown. **C.** Unpaired neuropeptide (top) and neuropeptide receptor (bottom) transcript abundance in 5 to 10 days post-emergence (dpe) non-blood fed (nbf; green) and age-matched blood fed (bf; brown) female antennae. Comparisons between nbf (black) and age-matched bf (blue) are described by fold change. Ball and stick diagrams represent the two general trends in abundance demonstrated by these gene families (motifs 1 and 2; bottom right). Asterisks between two age groups denote significant difference (> 2-fold change; FDR *P* < 0.05). Asterisks to the far right of each table indicate significant differences between 5 and 10 dpe. Above the horizontal grey line are the transcripts with an overall abundance greater than 1 TPM.

**Additional file 11: Figure S11**. Biogenic amine synthesis enzyme, receptor and transporter transcript abundance is age- and state-dependent. Synthesis of dopamine, octopamine and serotonin is depicted diagrammatically (**A**; left). Biogenic amine synthesis enzyme, receptor (**A**) and transporter (**B**) transcript abundance in 5 to 10 days post-emergence (dpe) non-blood fed (nbf; green; left) and age-matched blood fed (bf; brown; middle) *Aedes aegypti* female antennae. Comparisons between nbf (black) and age-matched bf (blue) are described by fold change (right). Permanent gene identifiers along with the common gene names are to the left. Ball and stick diagrams represent the two general trends in abundance demonstrated by these gene families (motifs 1 and 2; bottom right). Asterisks between two age groups denote significant difference (> 2-fold change; FDR *P* < 0.05). Asterisks to the far right of each table indicate significant differences between 5 and 10 dpe. Above the horizontal grey line are the transcripts with an overall abundance greater than 1 TPM.

**Additional file 12: Figure S12**. Acetylcholine synthesis enzyme, receptor and transporter transcript abundance is age- and state-dependent. Synthesis and recycling of acetylcholine is depicted diagrammatically (left). Acetylcholine synthesis enzyme, receptor and transporter transcript abundance in 5 to 10 days post-emergence (dpe) non-blood fed (nbf; green; left) and age-matched blood fed (bf; brown; middle) *Aedes aegypti* female antennae. Comparisons between nbf (black) and age-matched bf (blue) are described by fold change (right). Permanent gene identifiers along with the common gene names are to the left. Ball and stick diagrams represent the two general trends in abundance demonstrated by these gene families (motifs 1 and 2; bottom right). Asterisks between two age groups denote significant difference (> 2-fold change; FDR *P* < 0.05). Asterisks to the far right of each table indicate significant differences between 5 and 10 dpe. Above the horizontal grey line are the transcripts with an overall abundance greater than 1 TPM.

**Additional file 13: Figure S13**. Glutamate and GABA synthesis enzyme, receptor and transporter transcript abundance is age- and state-dependent. Synthesis and recycling of glutamate and GABA is depicted diagrammatically (left). Glutamate and GABA synthesis enzyme, receptor and transporter transcript abundance in 5 to 10 days post-emergence (dpe) non-blood fed (nbf; green; left) and age-matched blood fed (bf; brown; middle) *Aedes aegypti* female antennae. Comparisons between nbf (black) and age-matched bf (blue) are described by fold change (right). Permanent gene identifiers along with the common gene names are to the left. Ball and stick diagrams represent the two general trends in abundance demonstrated by these gene families (motifs 1 and 2; bottom right). Asterisks between two age groups denote significant difference (> 2-fold change; FDR *P* < 0.05). Asterisks to the far right of each table indicate significant differences between 5 and 10 dpe. Above the horizontal grey line are the transcripts with an overall abundance greater than 1 TPM.

**Additional file 14: Figure S14**. Transcription factor transcript abundance is age- and state-dependent. Transcription factor transcript abundance in 5 to 10 days post-emergence (dpe) non-blood fed (nbf; green; left) and age-matched blood fed (bf; brown; middle) *Aedes aegypti* female antennae. Comparisons between nbf (black) and age-matched bf (blue) are described by fold change (right). Permanent gene identifiers along with the common gene names are to the left. Ball and stick diagrams represent the two general trends in abundance demonstrated by this gene family (motifs 1 and 2; bottom right). Asterisks between two age groups denote significant difference (> 2-fold change; FDR *P* < 0.05). Asterisks to the far right of each table indicate significant differences between 5 and 10 dpe. Above the horizontal grey line are the transcripts with an overall abundance greater than 1 TPM.

**Additional file 15: Figure S15**. Abundance of core eukaryotic gene (CEG) transcripts. A comparison of fold change between the CEG antennal transcripts of non-blood fed and blood fed females among different age groups (5–10 days post-emergence).

**Additional file 16: Figure S16**. Abundance of core eukaryotic gene (CEG) transcripts. Pairwise comparisons of the CEG transcript abundance log_2_ fold change between each of the replicates for both states (non-blood fed and blood fed females) across the different age groups (5–10 days post-emergence).

**Additional file 17: Dataset S1.** Table of antennal transcript abundance (in TPM) for all ages of non-blood-fed and blood fed female *Aedes aegypti* (*Dataset S1 (TPM)*). Fold change and significance (FDR *p*-value) for comparisons of ages, and for comparisons of non-blood-fed and blood fed of matched ages (*Dataset S1 (TPM, FC, FDR P)*).

**Additional file 18: Dataset S2.** Table of antennal core eukaryotic gene transcript abundance (in TPM), fold change and significance (FDR p-value) for all ages of non-blood-fed and blood fed female *Aedes aegypti*, and for age-matched comparisons among states.

## Data Availability

All data generated or analyzed during this study are included in this published article and its supplementary information files. The transcriptomes generated and analysed during the current study are available in the NCBI project database, BioProject, with the ID PRJNA683174.
