# Supplementary figures and images for: Regulation of the antennal transcriptome of the dengue vector, Aedes aegypti, during the first gonotrophic cycle
Source: BMC Genomics. 2021 Jan 21;22:71. doi: 10.1186/s12864-020-07336-w (PMC7821643; doi:10.1186/s12864-020-07336-w)

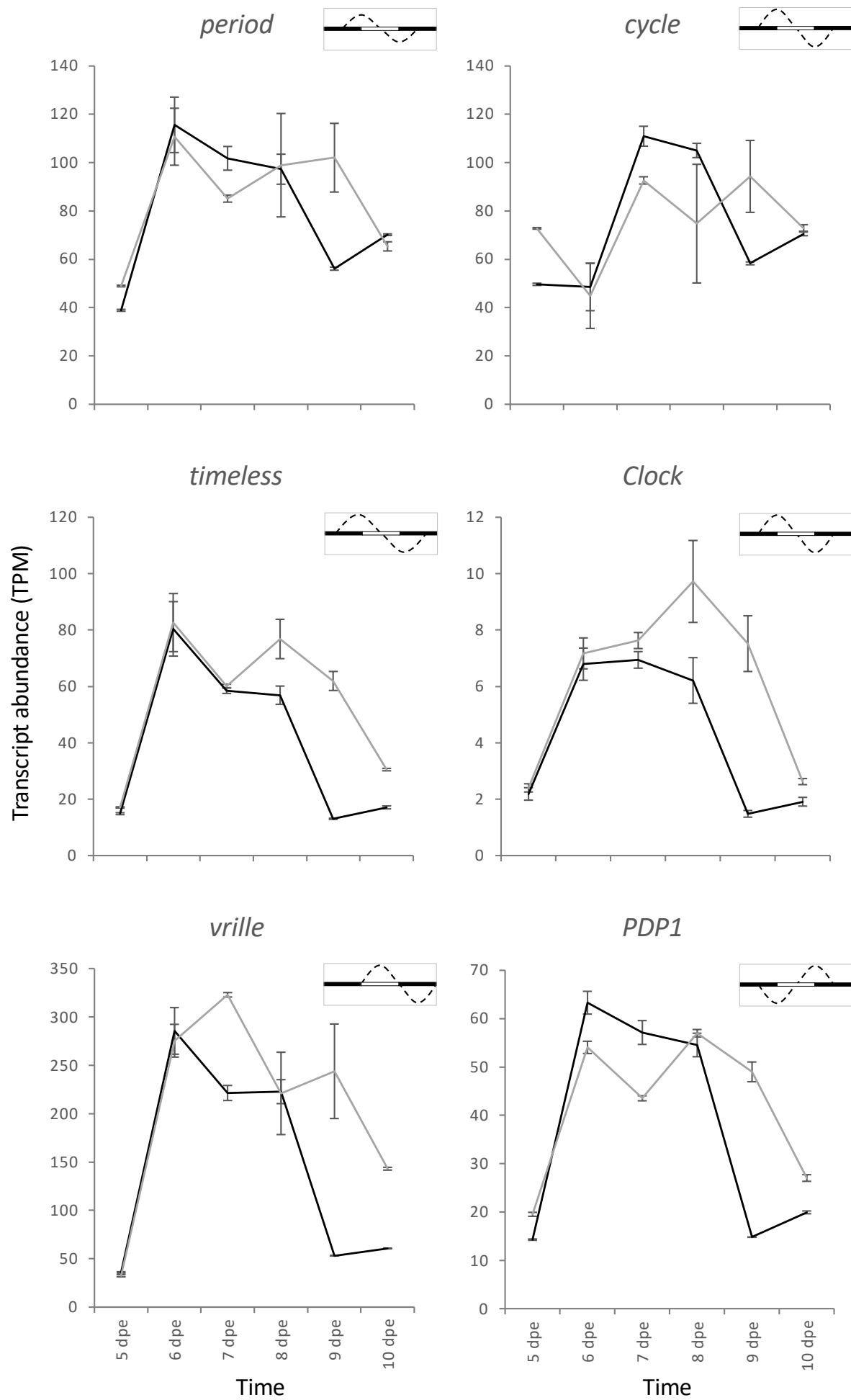

Supplement: Supplementary file 1 — Additional file 1: Figure S1. Abundance of the clock gene transcripts. Transcript abundances of period, clock, timeless, vrille, cycle and Pdp1 in the antenna of non-blood fed (black solid line) and blood fed (grey solid line) Aedes aegypti throughout the first gonotrophic cycle (5 to 10 days post-emergence; dpe). The error bars represent the standard error of the mean. [file 12864_2020_7336_MOESM1_ESM.pdf]

A. 5 dpe

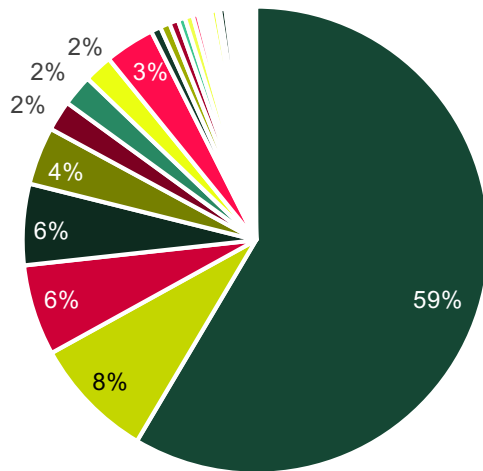

B. 6 dpe

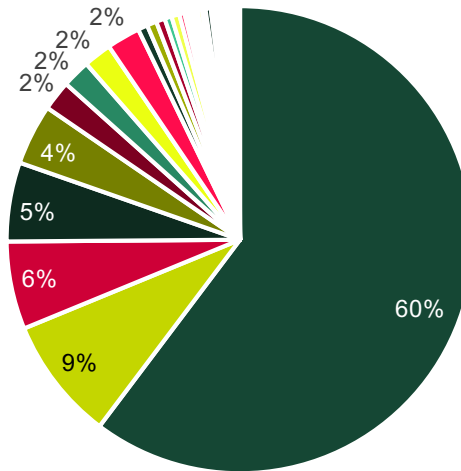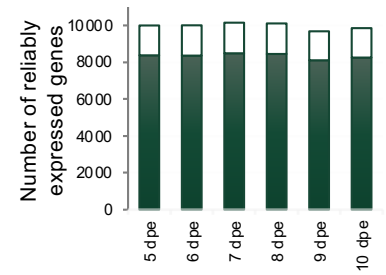

C. 7 dpe

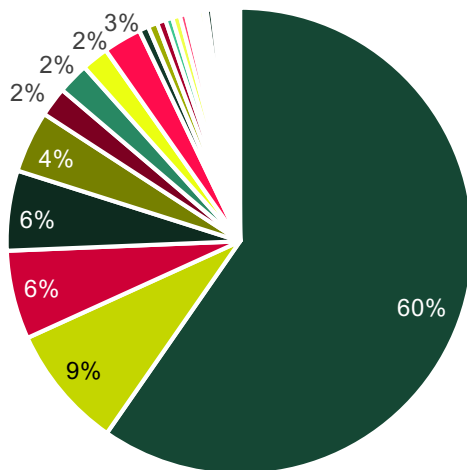

D. 8 dpe

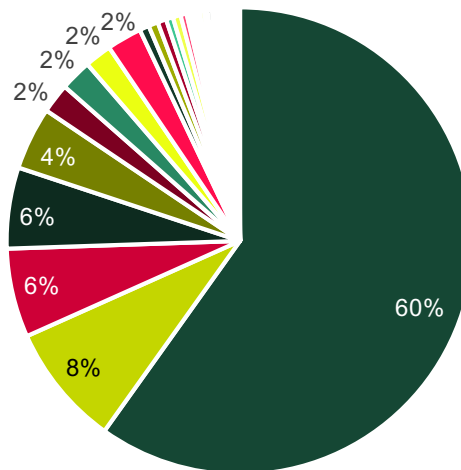

E. 9 dpe

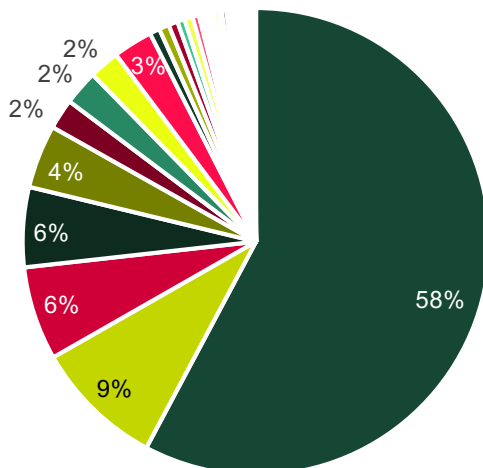

F. 10 dpe

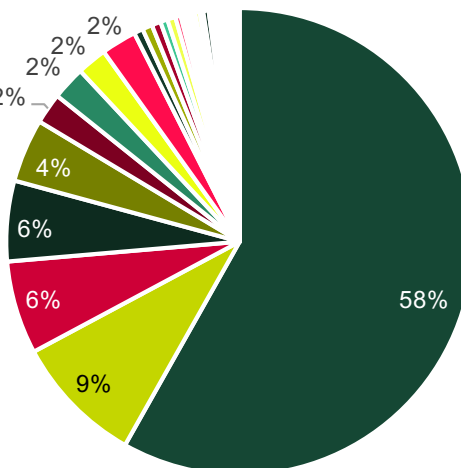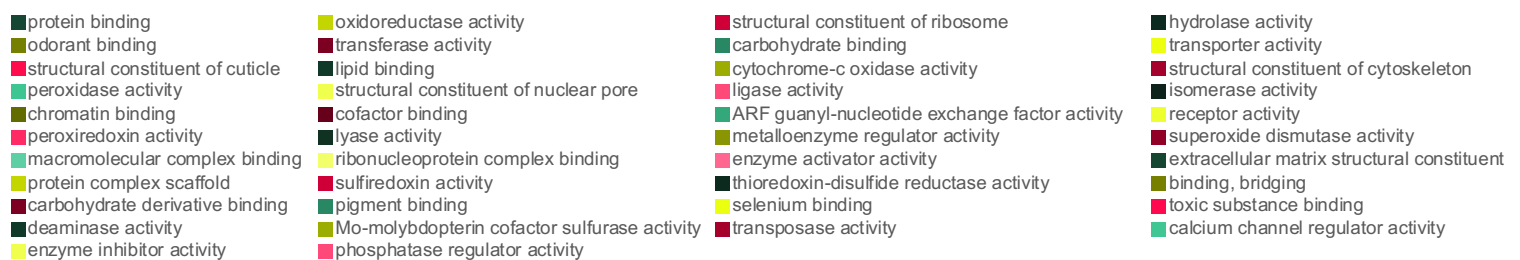

Supplement: Supplementary file 2 — Additional file 2: Figure S2. Reliable expression of antennal genes by molecular function. Proportions of reliably detected genes in the antennae of 5 to 10 days post-emergence (dpe) host-seeking adult female Aedes aegypti classified by a level 3 molecular function gene ontology (A-F). Inset: Total number of reliably detected genes from each age group can be determined by the sum of those with GO annotation (white) and those without (green). [file 12864_2020_7336_MOESM2_ESM.pdf]

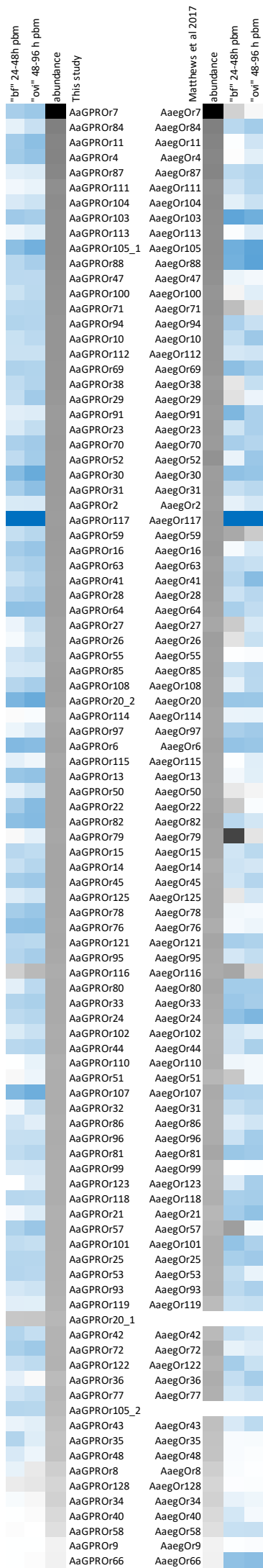

Supplement: Supplementary file 3 — Additional file 3: Figure S3. Comparison of odorant receptor abundance between this study and Matthews et al. (2016) [21]. The sugar fed condition in our data set is calculated as the average of all ages and replicates of nbf. The “bf” is calculated as the average of all bf replicates of 24 and 48 h pbm. The “ovi” is calculated as the average of all of the replicates from 48, 72 and 96 h pbm. These decisions were made based on the similarities with the described protocol in Matthews et al. 2016 [21]. Please note that there were 36 previously identified odorant receptor (Or) genes that are no longer included, or have been collapsed into variants of other Ors, in the present L5 annotation. Additionally, Or20 and Or115 are represented in the current L5 annotation by two genes each. [file 12864_2020_7336_MOESM3_ESM.pdf]

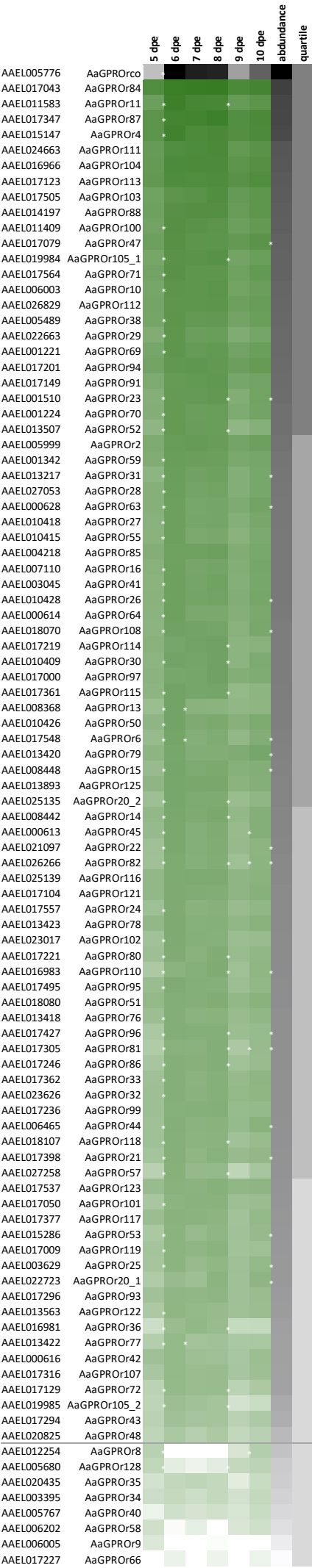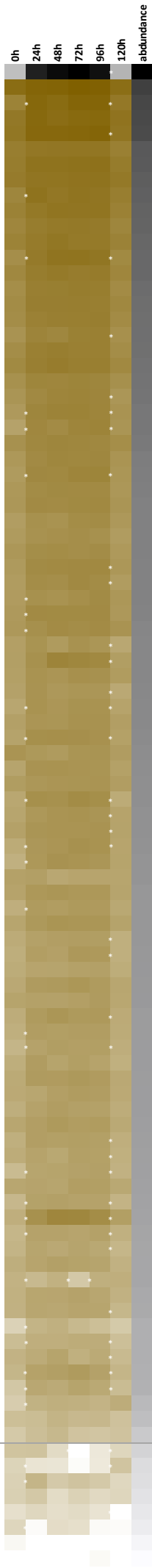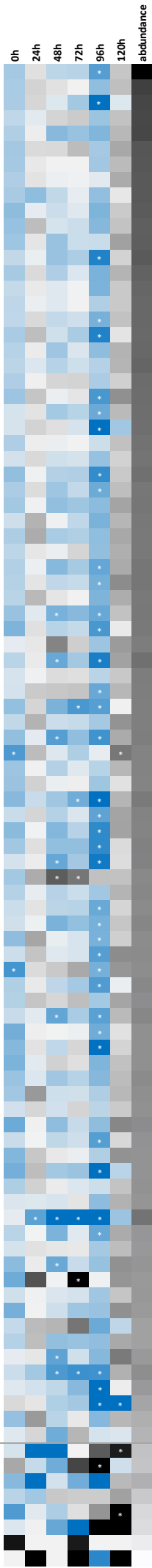

Supplement: Supplementary file 4 — Additional file 4: Figure S4. Odorant receptor transcript abundance is age- and state-dependent. Odorant receptor transcript abundance in 5 to 10 days post-emergence (dpe) non-blood fed (nbf; green; left) and age-matched blood fed (bf; brown; middle) Aedes aegypti female antennae. Comparisons between nbf (black) and age-matched bf (blue) antennal transcript abundance are described by fold change (right). Permanent gene identifiers along with the common gene names are to the left. Ball and stick diagrams represent the general trend in abundance demonstrated by this gene family (motif 1; bottom right). Asterisks between two age groups denote significant difference (> 2-fold change; FDR P < 0.05). Asterisks to the far right of each table indicate significant differences between 5 and 10 dpe. Above the horizontal grey line are the transcripts with an overall abundance greater than 1 TPM. [file 12864_2020_7336_MOESM4_ESM.pdf]

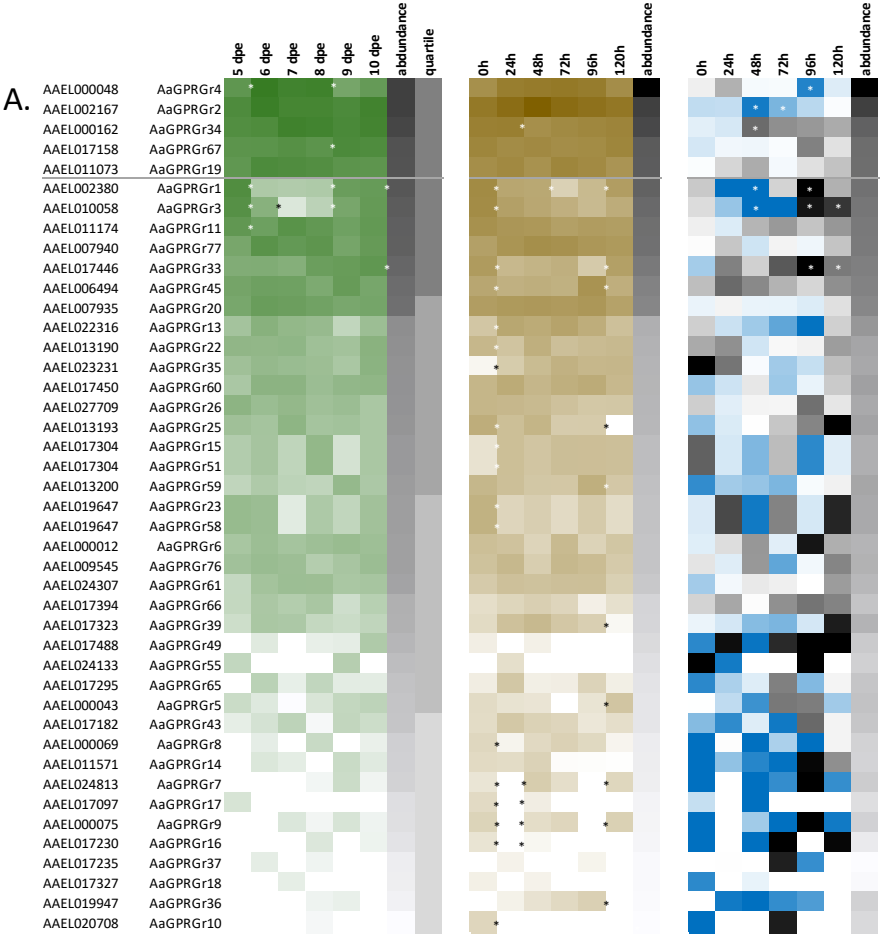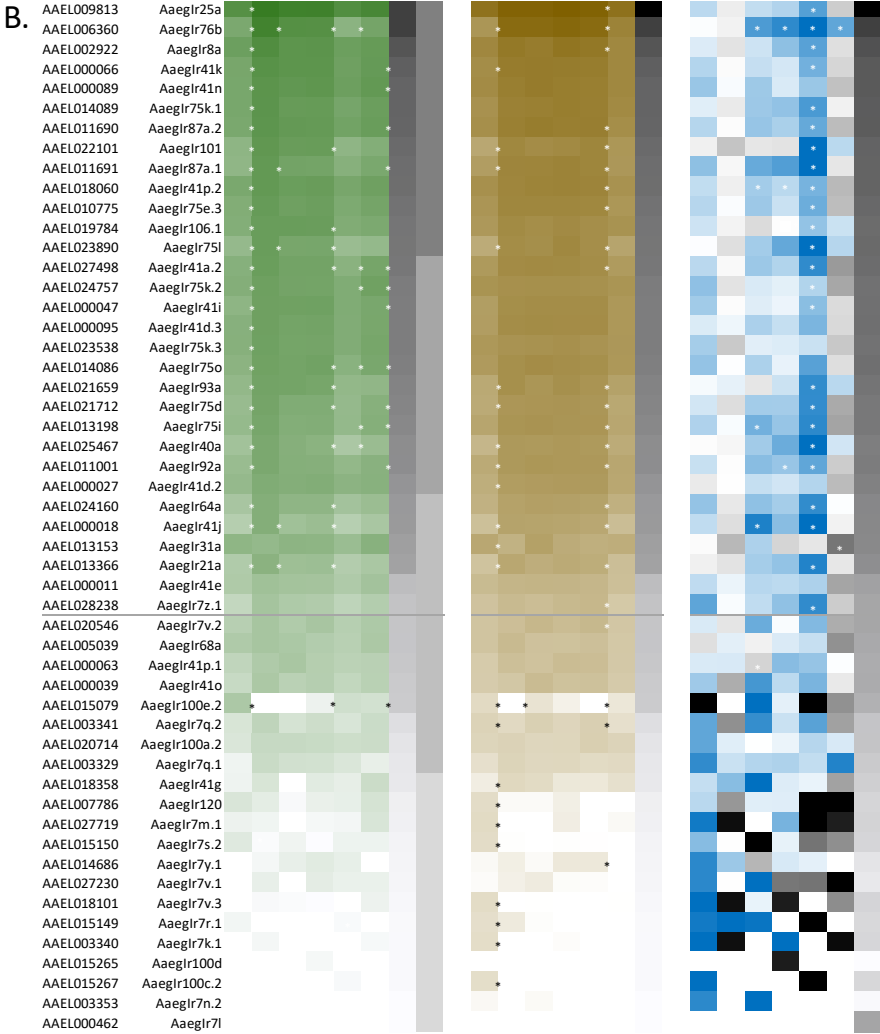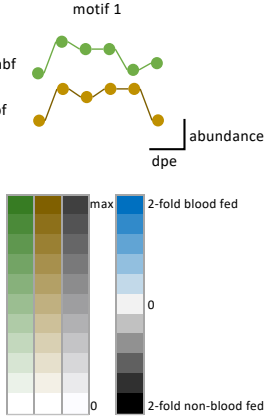

Supplement: Supplementary file 5 — Additional file 5: Figure S5. Gustatory and ionotropic receptor transcript abundance is age- and state-dependent. Gustatory (A) and ionotropic (B) receptor transcript abundance in 5 to 10 days post-emergence (dpe) non-blood fed (nbf; green; left) and age-matched blood fed (bf; brown; middle) Aedes aegypti female antennae. Comparisons between nbf (black) and age-matched bf (blue) are described by fold change (right). Permanent gene identifiers along with the common gene names are to the left. Ball and stick diagrams represent the general trend in abundance demonstrated by these gene families (motif 1; bottom right). Asterisks between two age groups denote significant difference (> 2-fold change; FDR P < 0.05). Asterisks to the far right of each table indicate significant differences between 5 and 10 dpe. Above the horizontal grey line are the transcripts with an overall abundance greater than 1 TPM. [file 12864_2020_7336_MOESM5_ESM.pdf]

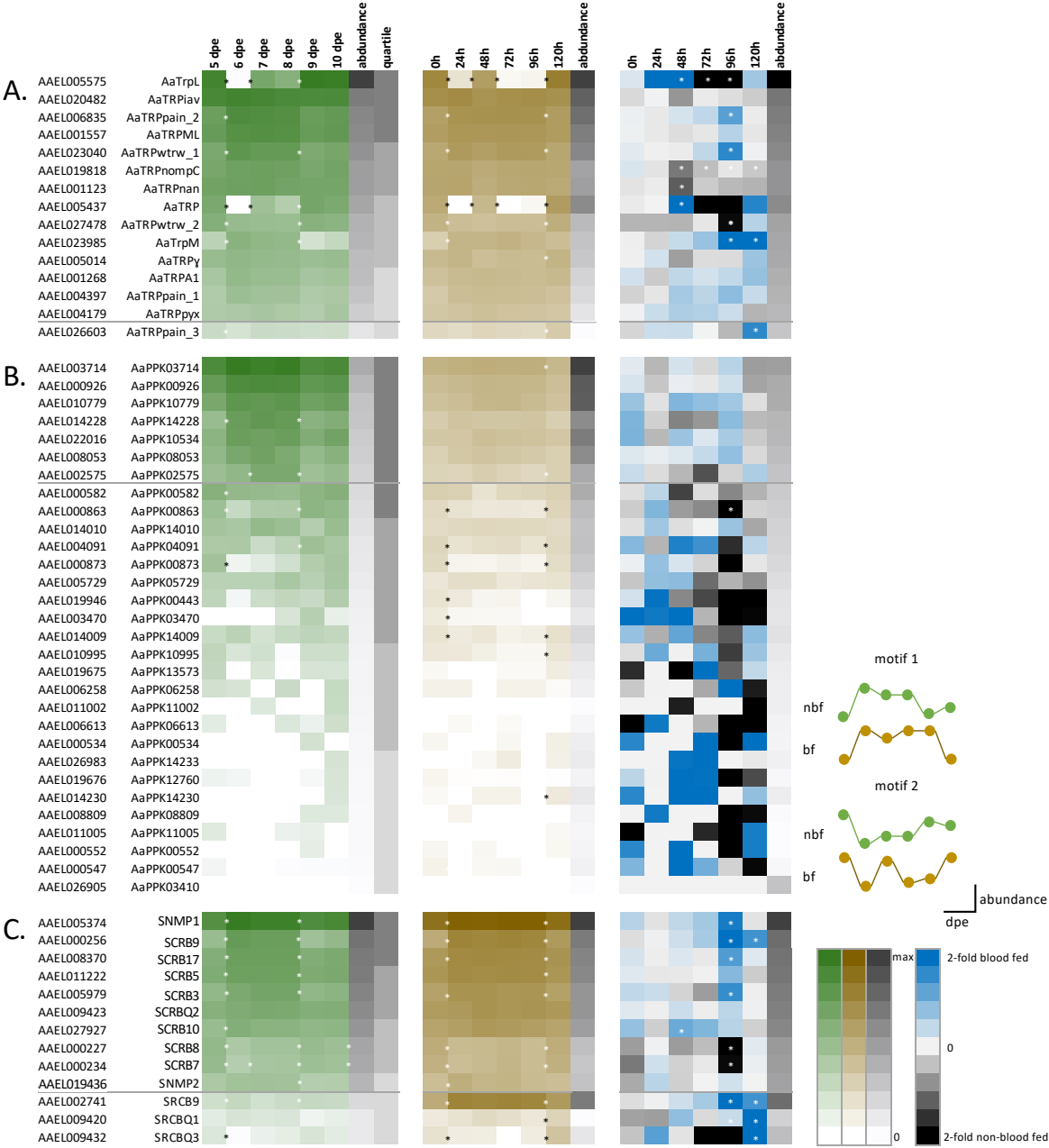

Supplement: Supplementary file 6 — Additional file 6: Figure S6. Transient receptor potential, pickpocket and class B scavenger receptor transcript abundance is age- and state-dependent. Transient receptor potential (A), pickpocket (B) and class B scavenger (C) receptor transcript abundance in 5 to 10 days post-emergence (dpe) non-blood fed (nbf; green; left) and age-matched blood fed (bf; brown; middle) Aedes aegypti female antennae. Comparisons between nbf (black) and age-matched bf (blue) are described by fold change (right). Permanent gene identifiers along with the common gene names are to the left. Ball and stick diagrams represent the two general trends in abundance demonstrated by these gene families (motifs 1 and 2; bottom right). Asterisks between two age groups denote significant difference (> 2-fold change; FDR P < 0.05). Asterisks to the far right of each table indicate significant differences between 5 and 10 dpe. Above the horizontal grey line are the transcripts with an overall abundance greater than 1 TPM. [file 12864_2020_7336_MOESM6_ESM.pdf]

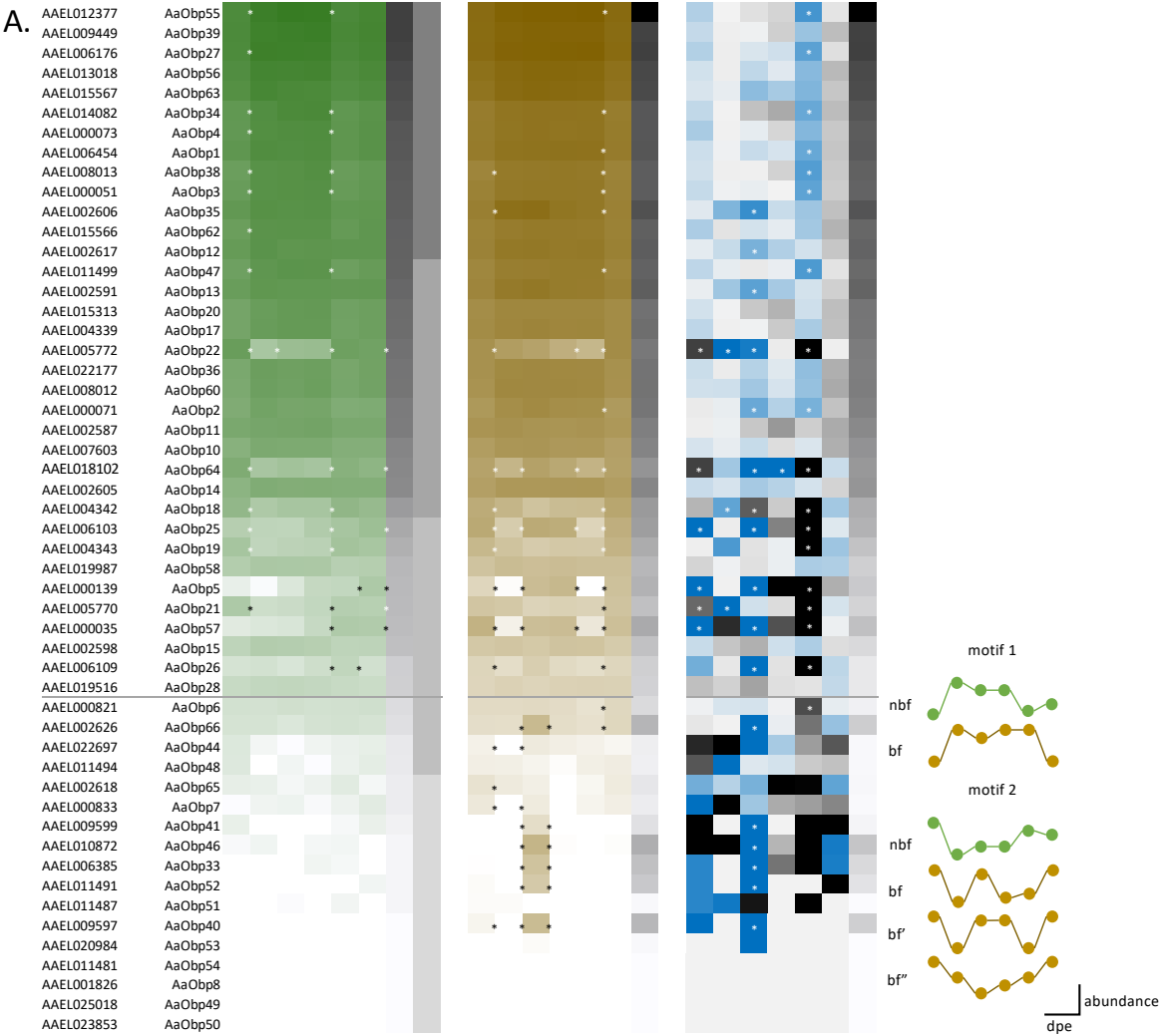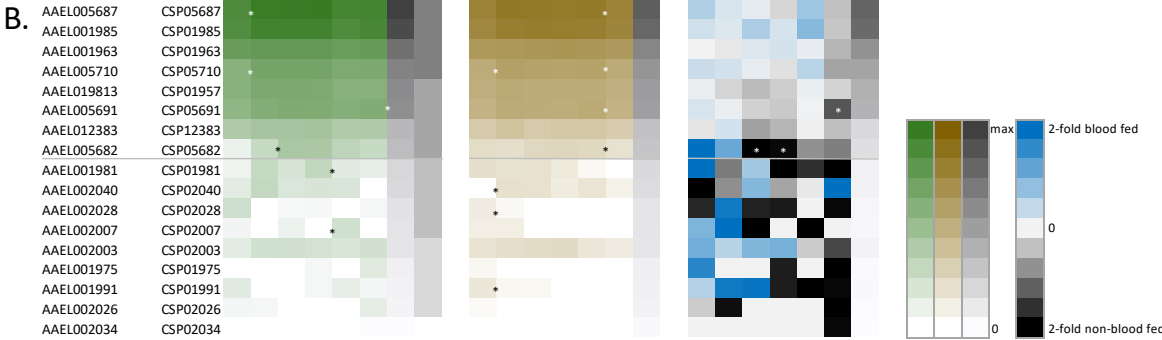

Supplement: Supplementary file 7 — Additional file 7: Figure S7. Odorant binding protein and chemosensory protein transcript abundance is age- and state-dependent. Odorant binding protein (A) and chemosensory protein (B) transcript abundance in 5 to 10 days post-emergence (dpe) non-blood fed (nbf; green; left) and age-matched blood fed (bf; brown; middle) Aedes aegypti female antennae. Comparisons between nbf (black) and age-matched bf (blue) are described by fold change (right). Permanent gene identifiers along with the common gene names are to the left. Ball and stick diagrams represent the two general trends in abundance demonstrated by these gene families (motifs 1 and 2; bottom right). Asterisks between two age groups denote significant difference (> 2-fold change; FDR P < 0.05). Asterisks to the far right of each table indicate significant differences between 5 and 10 dpe. Above the horizontal grey line are the transcripts with an overall abundance greater than 1 TPM. [file 12864_2020_7336_MOESM7_ESM.pdf]

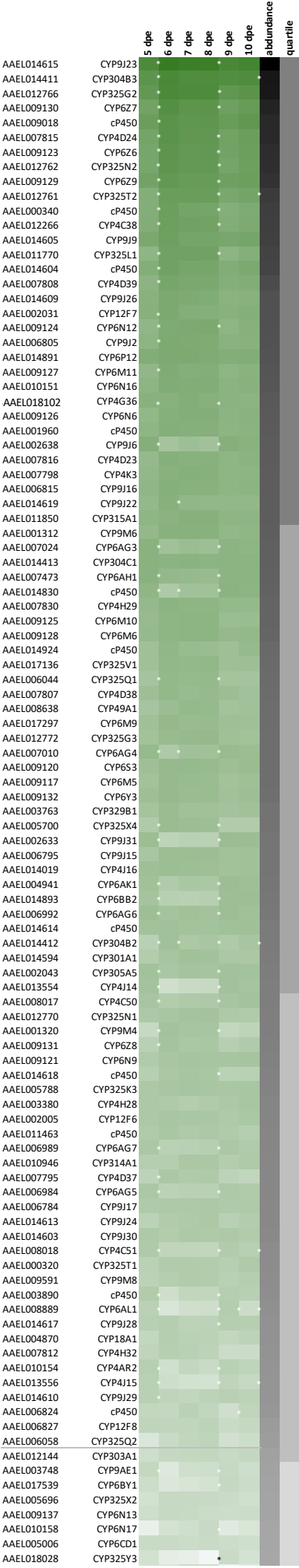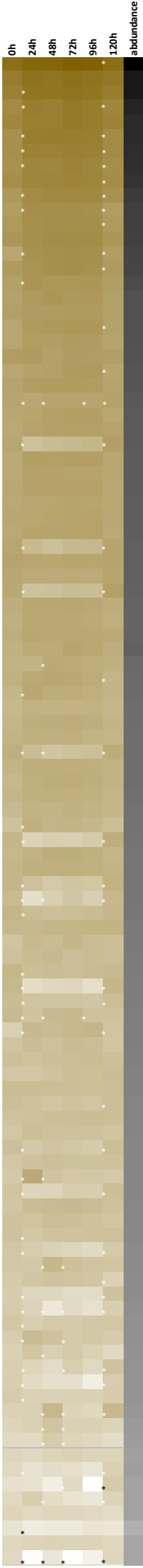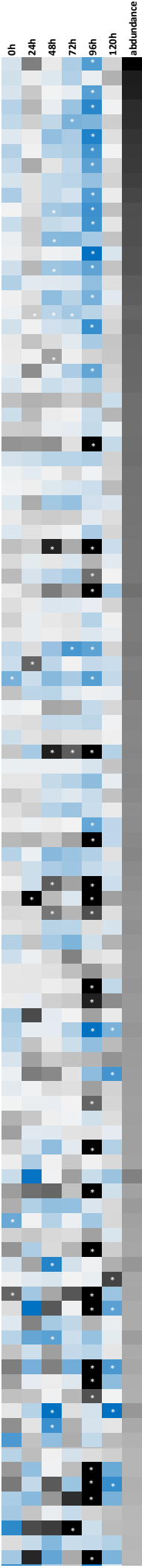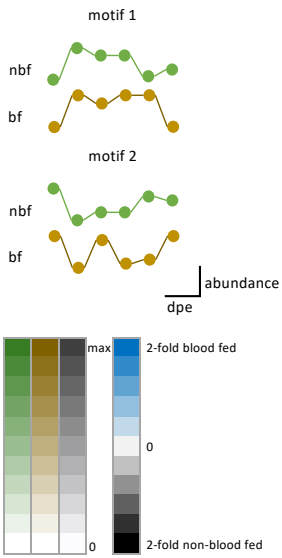

Supplement: Supplementary file 8 — Additional file 8: Figure S8. Cytochrome P450 oxidase transcript abundance is age- and state-dependent. Cytochrome P450 oxidase transcript abundance in 5 to 10 days post-emergence (dpe) non-blood fed (nbf; green; left) and age-matched blood fed (bf; brown; middle) Aedes aegypti female antennae. Comparisons between nbf (black) and age-matched bf (blue) are described by fold change (right). Permanent gene identifiers along with the common gene names are to the left. Ball and stick diagrams represent the two general trends in abundance demonstrated by this gene family (motifs 1 and 2; bottom right). Asterisks between two age groups denote significant difference (> 2-fold change; FDR P < 0.05). Asterisks to the far right of each table indicate significant differences between 5 and 10 dpe. Above the horizontal grey line are the transcripts with an overall abundance greater than 1 TPM. [file 12864_2020_7336_MOESM8_ESM.pdf]

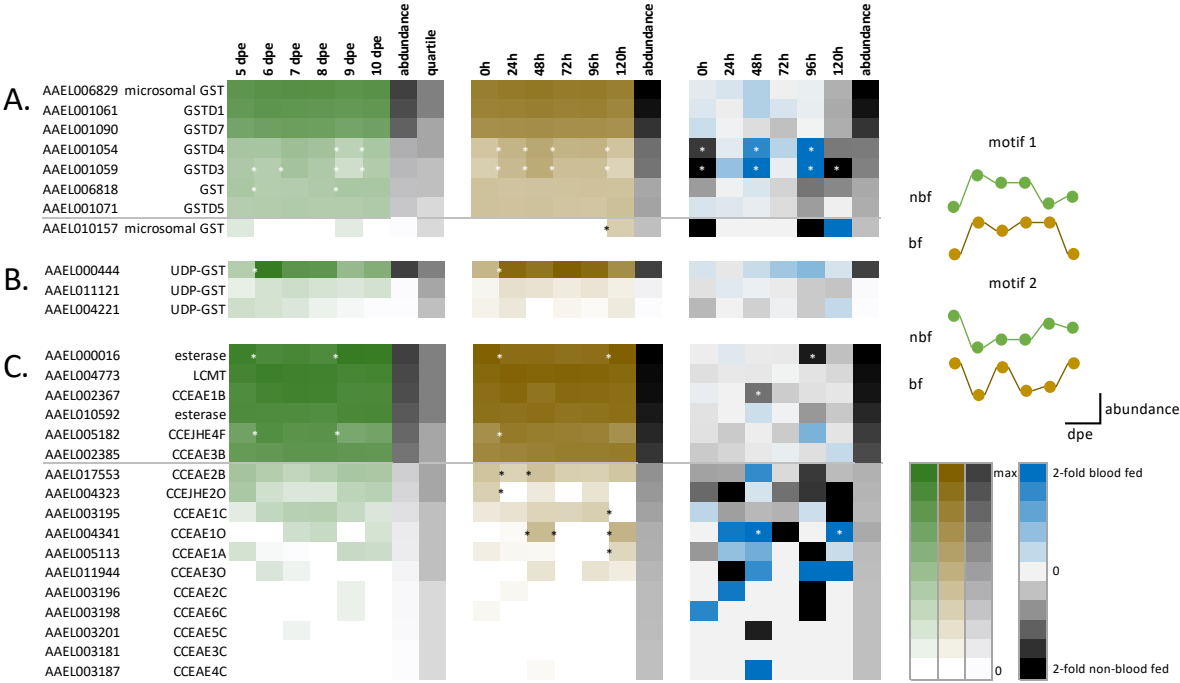

Supplement: Supplementary file 9 — Additional file 9: Figure S9. Glutathione-S-transferase, UDP-glucosyltransferase and carboxyl/ cholinesterase transcript abundance is age- and state-dependent. Glutathione-S-transferase (GST; A), UDP-glucosyltransferase (UDP-GST; B) and carboxyl/cholinesterase (CCE; C) transcript abundance in 5 to 10 days post-emergence (dpe) non-blood fed (nbf; green; left) and age-matched blood fed (bf; brown; middle) Aedes aegypti female antennae. Comparisons between nbf (black) and age-matched bf (blue) are described by fold change (right). Permanent gene identifiers along with the common gene names are to the left. Ball and stick diagrams represent the two general trends in abundance demonstrated by these gene families (motifs 1 and 2; bottom right). Asterisks between two age groups denote significant difference (> 2-fold change; FDR P < 0.05). Above the horizontal grey line are the transcripts with an overall abundance greater than 1 TPM. [file 12864_2020_7336_MOESM9_ESM.pdf]

## B. Transporters

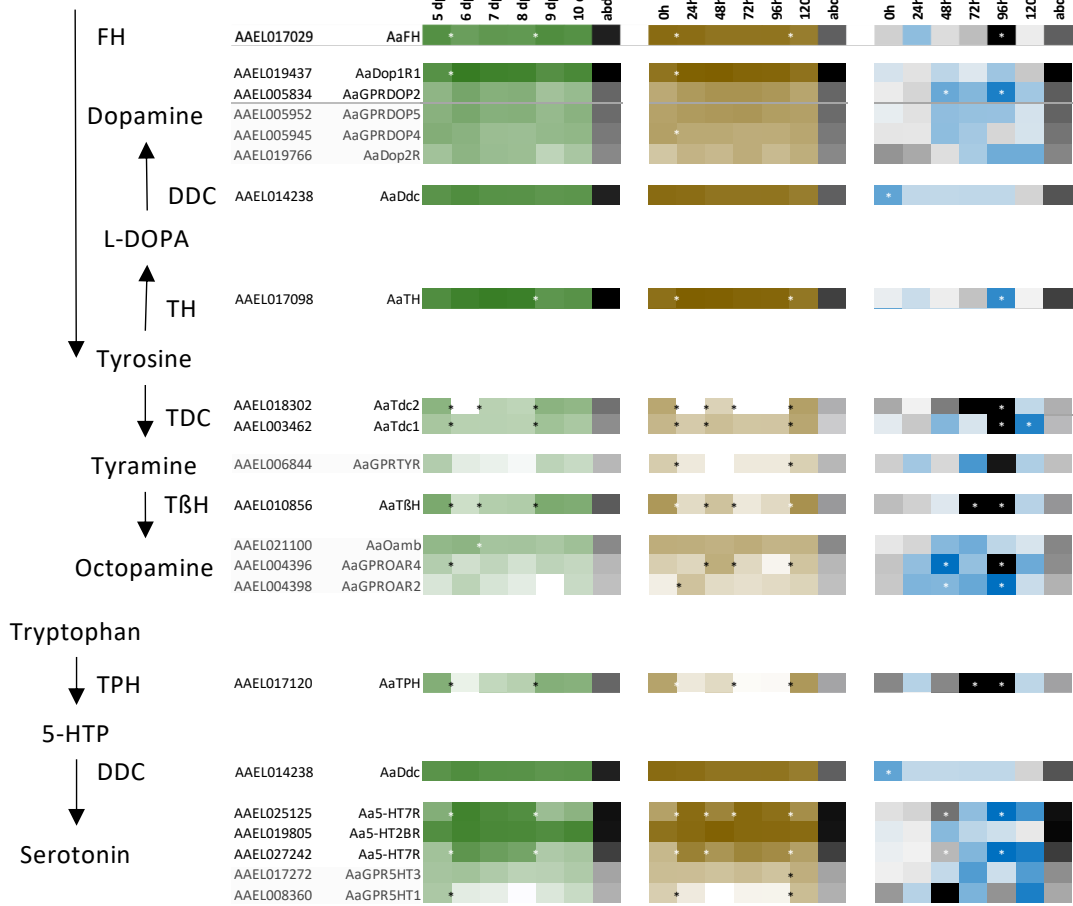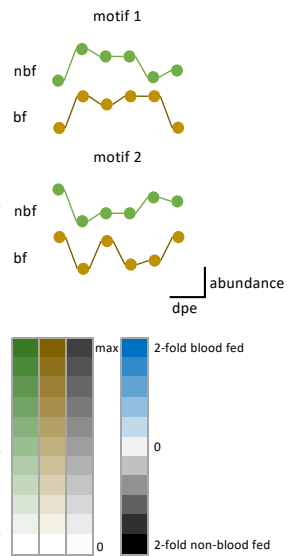

## B. Transporters

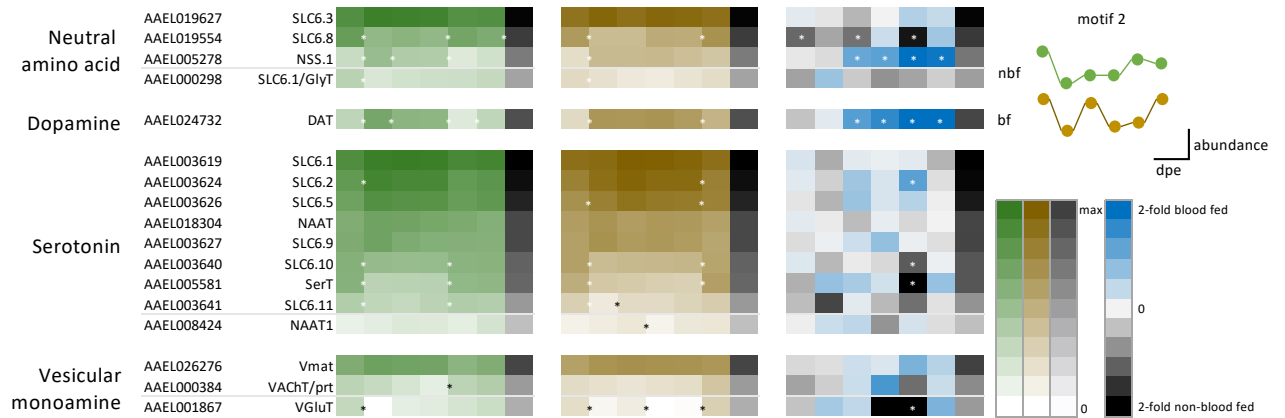

Supplement: Supplementary file 11 — Additional file 11: Figure S11. Biogenic amine synthesis enzyme, receptor and transporter transcript abundance is age- and state-dependent. Synthesis of dopamine, octopamine and serotonin is depicted diagrammatically (A; left). Biogenic amine synthesis enzyme, receptor (A) and transporter (B) transcript abundance in 5 to 10 days post-emergence (dpe) non-blood fed (nbf; green; left) and age-matched blood fed (bf; brown; middle) Aedes aegypti female antennae. Comparisons between nbf (black) and age-matched bf (blue) are described by fold change (right). Permanent gene identifiers along with the common gene names are to the left. Ball and stick diagrams represent the two general trends in abundance demonstrated by these gene families (motifs 1 and 2; bottom right). Asterisks between two age groups denote significant difference (> 2-fold change; FDR P < 0.05). Asterisks to the far right of each table indicate significant differences between 5 and 10 dpe. Above the horizontal grey line are the transcripts with an overall abundance greater than 1 TPM. [file 12864_2020_7336_MOESM11_ESM.pdf]

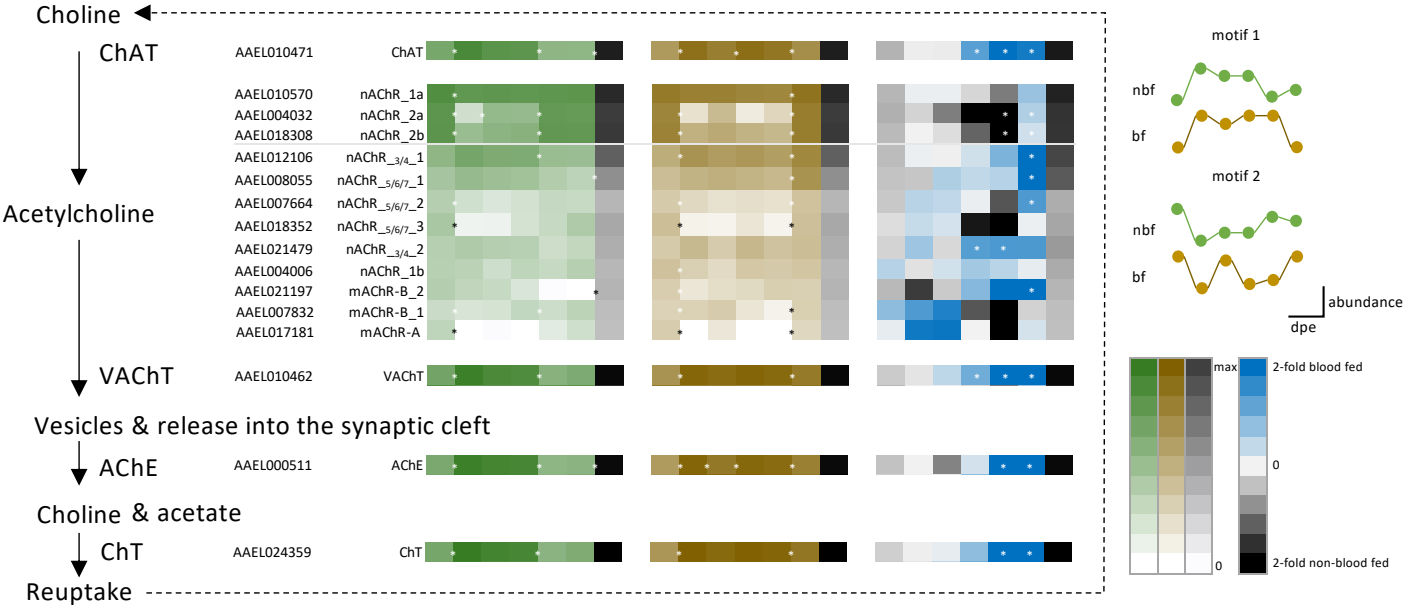

Supplement: Supplementary file 12 — Additional file 12: Figure S12. Acetylcholine synthesis enzyme, receptor and transporter transcript abundance is age- and state-dependent. Synthesis and recycling of acetylcholine is depicted diagrammatically (left). Acetylcholine synthesis enzyme, receptor and transporter transcript abundance in 5 to 10 days post-emergence (dpe) non-blood fed (nbf; green; left) and age-matched blood fed (bf; brown; middle) Aedes aegypti female antennae. Comparisons between nbf (black) and age-matched bf (blue) are described by fold change (right). Permanent gene identifiers along with the common gene names are to the left. Ball and stick diagrams represent the two general trends in abundance demonstrated by these gene families (motifs 1 and 2; bottom right). Asterisks between two age groups denote significant difference (> 2-fold change; FDR P < 0.05). Asterisks to the far right of each table indicate significant differences between 5 and 10 dpe. Above the horizontal grey line are the transcripts with an overall abundance greater than 1 TPM. [file 12864_2020_7336_MOESM12_ESM.pdf]

Glutamate reuptake

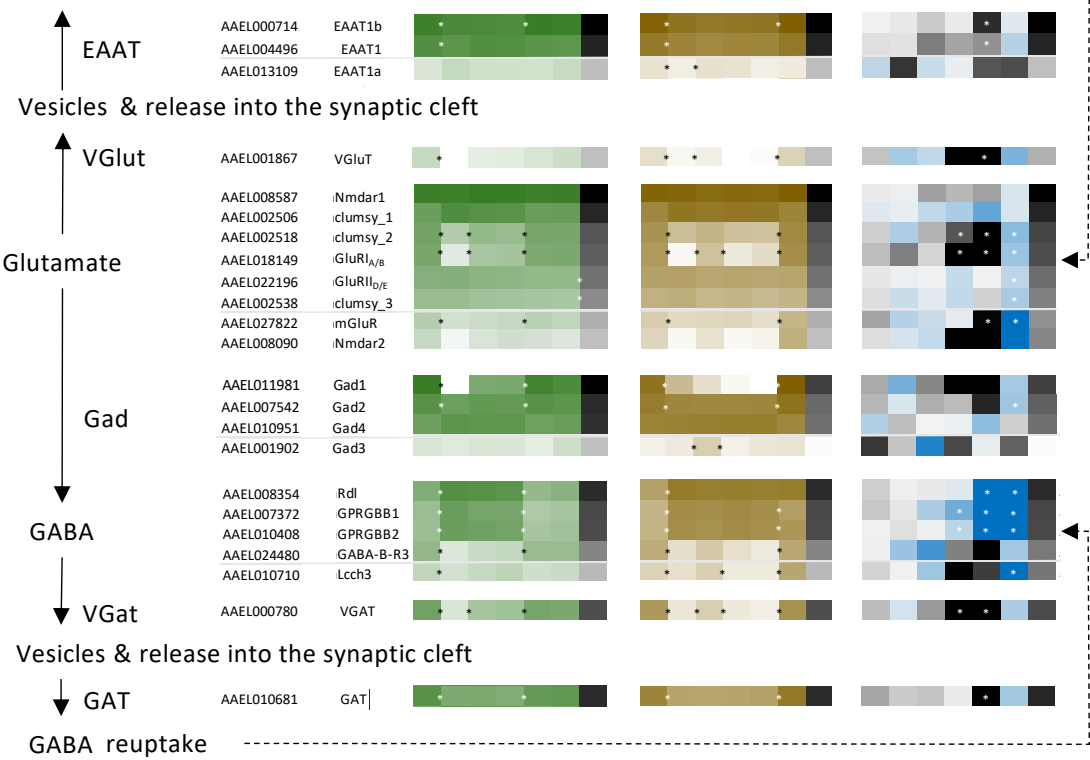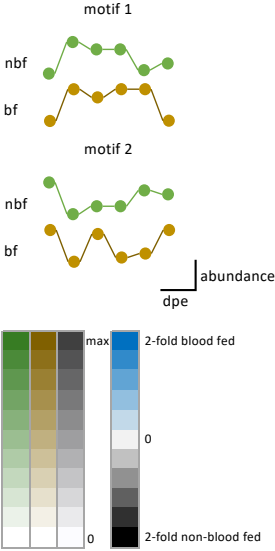

Supplement: Supplementary file 13 — Additional file 13: Figure S13. Glutamate and GABA synthesis enzyme, receptor and transporter transcript abundance is age- and state-dependent. Synthesis and recycling of glutamate and GABA is depicted diagrammatically (left). Glutamate and GABA synthesis enzyme, receptor and transporter transcript abundance in 5 to 10 days post-emergence (dpe) non-blood fed (nbf; green; left) and age-matched blood fed (bf; brown; middle) Aedes aegypti female antennae. Comparisons between nbf (black) and age-matched bf (blue) are described by fold change (right). Permanent gene identifiers along with the common gene names are to the left. Ball and stick diagrams represent the two general trends in abundance demonstrated by these gene families (motifs 1 and 2; bottom right). Asterisks between two age groups denote significant difference (> 2-fold change; FDR P < 0.05). Asterisks to the far right of each table indicate significant differences between 5 and 10 dpe. Above the horizontal grey line are the transcripts with an overall abundance greater than 1 TPM. [file 12864_2020_7336_MOESM13_ESM.pdf]

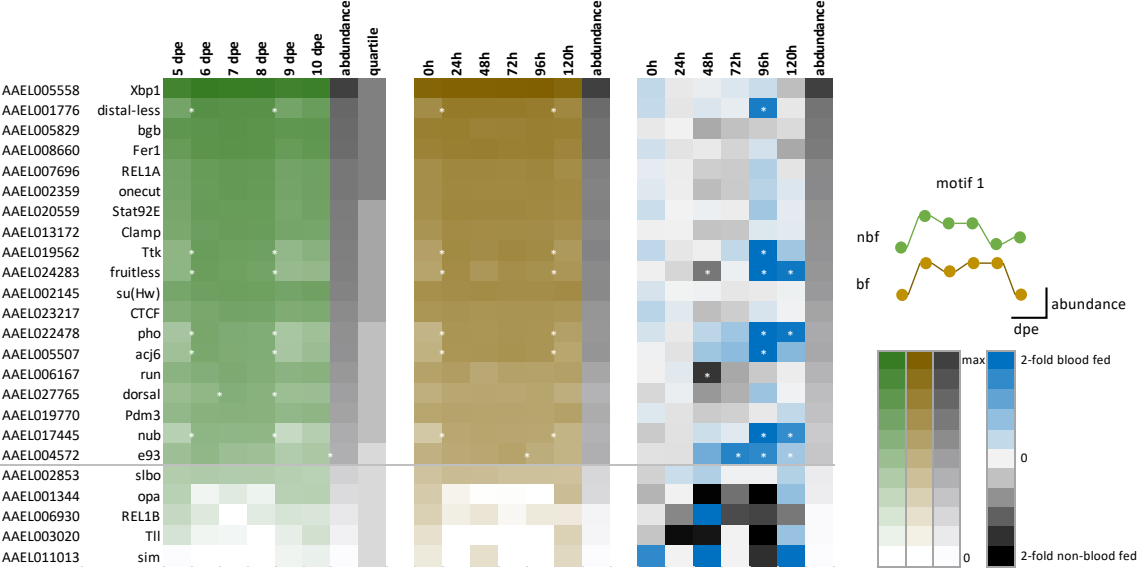

Supplement: Supplementary file 14 — Additional file 14: Figure S14. Transcription factor transcript abundance is age- and state-dependent. Transcription factor transcript abundance in 5 to 10 days post-emergence (dpe) non-blood fed (nbf; green; left) and age-matched blood fed (bf; brown; middle) Aedes aegypti female antennae. Comparisons between nbf (black) and age-matched bf (blue) are described by fold change (right). Permanent gene identifiers along with the common gene names are to the left. Ball and stick diagrams represent the two general trends in abundance demonstrated by this gene family (motifs 1 and 2; bottom right). Asterisks between two age groups denote significant difference (> 2-fold change; FDR P < 0.05). Asterisks to the far right of each table indicate significant differences between 5 and 10 dpe. Above the horizontal grey line are the transcripts with an overall abundance greater than 1 TPM. [file 12864_2020_7336_MOESM14_ESM.pdf]

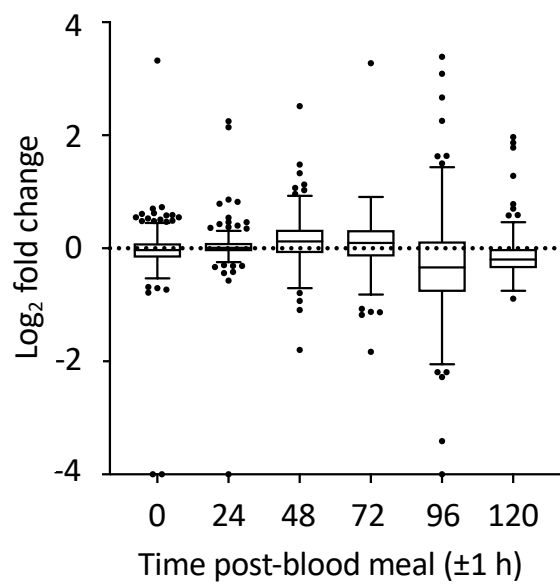

Supplement: Supplementary file 15 — Additional file 15: Figure S15. Abundance of core eukaryotic gene (CEG) transcripts. A comparison of fold change between the CEG antennal transcripts of non-blood fed and blood fed females among different age groups (5–10 days post-emergence). [file 12864_2020_7336_MOESM15_ESM.pdf]
