# Supplementary material for: Regulation of the antennal transcriptome of the dengue vector, Aedes aegypti, during the first gonotrophic cycle
Source: BMC Genomics. 2021 Jan 21;22:71. doi: 10.1186/s12864-020-07336-w (PMC7821643; doi:10.1186/s12864-020-07336-w)
Supplement: Supplementary file 10 — Additional file 10: Figure S10. Neuropeptide and neuropeptide receptor transcript abundance is age- and state-dependent. Paired neuropeptide (right) and neuropeptide receptor (left; A), transcript abundance in 5 to 10 days post-emergence (dpe) non-blood fed (nbf; green) and age-matched blood fed (bf; brown) Aedes aegypti female antennae. Comparisons between nbf (black) and age-matched bf (blue) are described by fold change. Permanent gene identifiers along with the common gene names are indicated. B. Identified modulators of insect gonotrophic behaviours, insulin-like peptide (ILP), short neuropeptide F (sNPF) and tachykinin (TK) peptides and their cognate receptors are shown. C. Unpaired neuropeptide (top) and neuropeptide receptor (bottom) transcript abundance in 5 to 10 days post-emergence (dpe) non-blood fed (nbf; green) and age-matched blood fed (bf; brown) female antennae. Comparisons between nbf (black) and age-matched bf (blue) are described by fold change. Ball and stick diagrams represent the two general trends in abundance demonstrated by these gene families (motifs 1 and 2; bottom right). Asterisks between two age groups denote significant difference (> 2-fold change; FDR P < 0.05). Asterisks to the far right of each table indicate significant differences between 5 and 10 dpe. Above the horizontal grey line are the transcripts with an overall abundance greater than 1 TPM. [file 12864_2020_7336_MOESM10_ESM.pdf]

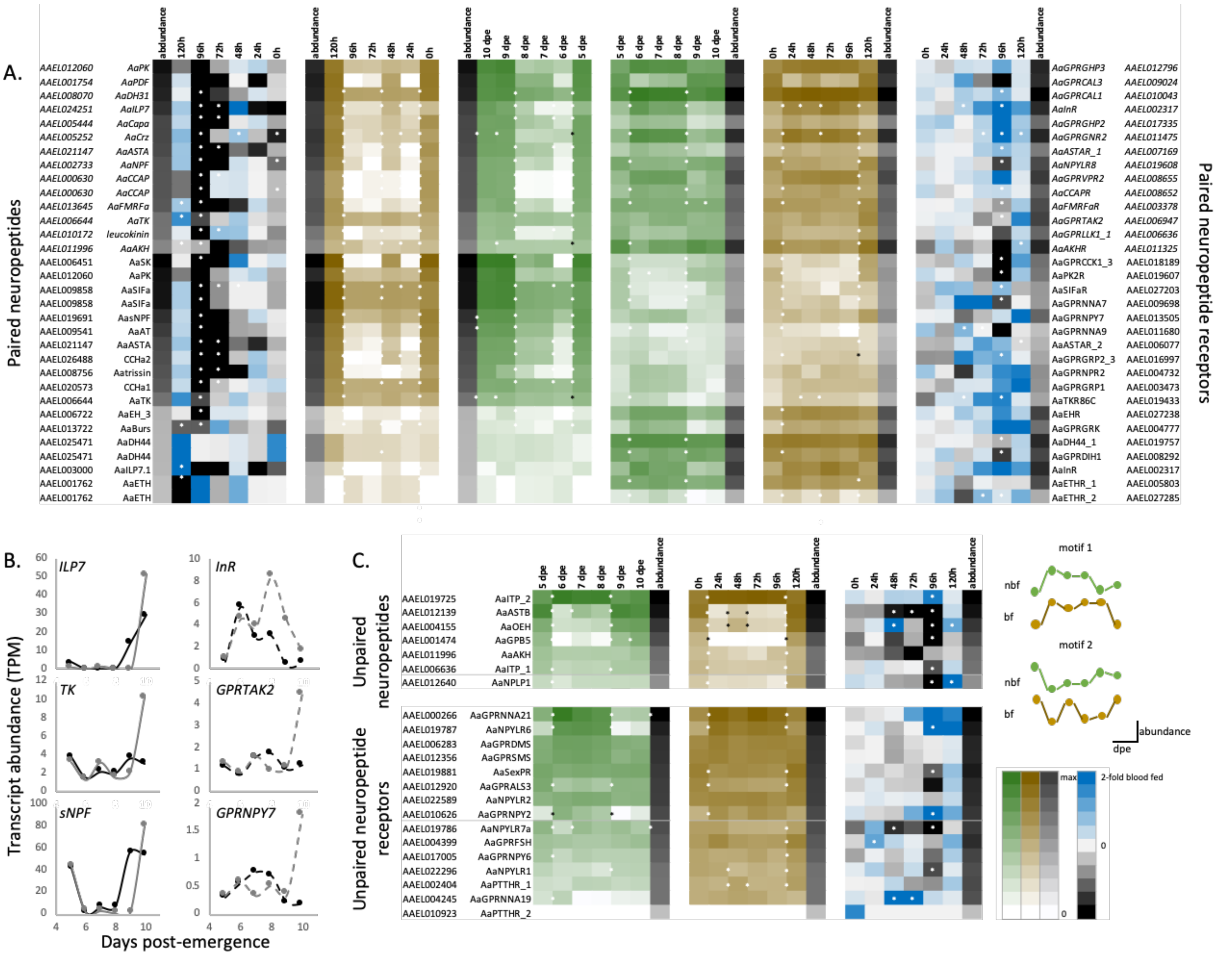

A.
Paired neuropeptides
Paired neuropeptide receptors
abundance
120h
96h
72h
48h
24h
0h
10 dpe
9 dpe
8 dpe
7 dpe
6 dpe
5 dpe
AAEL012060 AaPK
AAEL001754 AaPDF
AAEL008070 AaDH31
AAEL024251 AaILP7
AAEL005444 AaCapa
AAEL005252 AaCrz
AAEL021147 AaASTA
AAEL002733 AaNPF
AAEL000630 AaCCAP
AAEL000630 AaCCAP
AAEL013645 AaFMRFa
AAEL006644 AaTK
AAEL010172 leucokinin
AAEL011996 AaAKH
AAEL006451 AaSK
AAEL012060 AaPK
AAEL009858 AaSIFa
AAEL009858 AaSIFa
AAEL019691 AasNPF
AAEL009541 AaAT
AAEL021147 AaASTA
AAEL026488 CCHa2
AAEL008756 Aatrissin
AAEL020573 CCHa1
AAEL006644 AaTK
AAEL006722 AaEH_3
AAEL013722 AaBurs
AAEL025471 AaDH44
AAEL025471 AaDH44
AAEL003000 AaILP7.1
AAEL001762 AaETH
AAEL001762 AaETH
AaGPRGHP3 AAEL012796
AaGPRCAL3 AAEL009024
AaGPRCAL1 AAEL010043
AaInR AAEL002317
AaGPRGHP2 AAEL017335
AaGPRGNR2 AAEL011475
AaASTAR_1 AAEL007169
AaNPYLR8 AAEL019608
AaGPRVPR2 AAEL008655
AaCCAPR AAEL008652
AaFMRFaR AAEL003378
AaGPRTAK2 AAEL006947
AaGPRLLK1_1 AAEL006636
AaAKHR AAEL011325
AaGPRCCK1_3 AAEL018189
AaPK2R AAEL019607
AaSIFaR AAEL027203
AaGPRNNA7 AAEL009698
AaGPRNPY7 AAEL013505
AaGPRNNA9 AAEL011680
AaASTAR_2 AAEL006077
AaGPRGRP2_3 AAEL016997
AaGPRNPR2 AAEL004732
AaGPRGRP1 AAEL003473
AaTKR86C AAEL019433
AaEHR AAEL027238
AaGPRGRK AAEL004777
AaDH44_1 AAEL019757
AaGPRDIH1 AAEL008292
AaInR AAEL002317
AaETHR_1 AAEL005803
AaETHR_2 AAEL027285
B.
Transcript abundance (TPM)
Days post-emergence
ILP7
InR
TK
GPRTAK2
sNPF
GPRNPY7
C.
Unpaired neuropeptides
Unpaired neuropeptide receptors
AAEL019725 AaITP_2
AAEL012139 AaASTB
AAEL004155 AaOEH
AAEL001474 AaGPB5
AAEL011996 AaAKH
AAEL006636 AaITP_1
AAEL012640 AaNPLP1
AAEL000266 AaGPRNNA21
AAEL019787 AaNPYLR6
AAEL006283 AaGPRDMS
AAEL012356 AaGPRSMS
AAEL019881 AaSexPR
AAEL012920 AaGPRALS3
AAEL022589 AaNPYLR2
AAEL010626 AaGPRNPY2
AAEL019786 AaNPYLR7a
AAEL004399 AaGPRFSH
AAEL017005 AaGPRNPY6
AAEL022296 AaNPYLR1
AAEL002404 AaPTTHR_1
AAEL004245 AaGPRNNA19
AAEL010923 AaPTTHR_2
motif 1
motif 2
nbf
bf
abundance
dpe
max
2-fold blood fed
0
