# Supplementary material for: Regulation of the antennal transcriptome of the dengue vector, Aedes aegypti, during the first gonotrophic cycle
Source: BMC Genomics. 2021 Jan 21;22:71. doi: 10.1186/s12864-020-07336-w (PMC7821643; doi:10.1186/s12864-020-07336-w)
Supplement: Supplementary file 16 — Additional file 16: Figure S16. Abundance of core eukaryotic gene (CEG) transcripts. Pairwise comparisons of the CEG transcript abundance log2 fold change between each of the replicates for both states (non-blood fed and blood fed females) across the different age groups (5–10 days post-emergence). [file 12864_2020_7336_MOESM16_ESM.pdf]

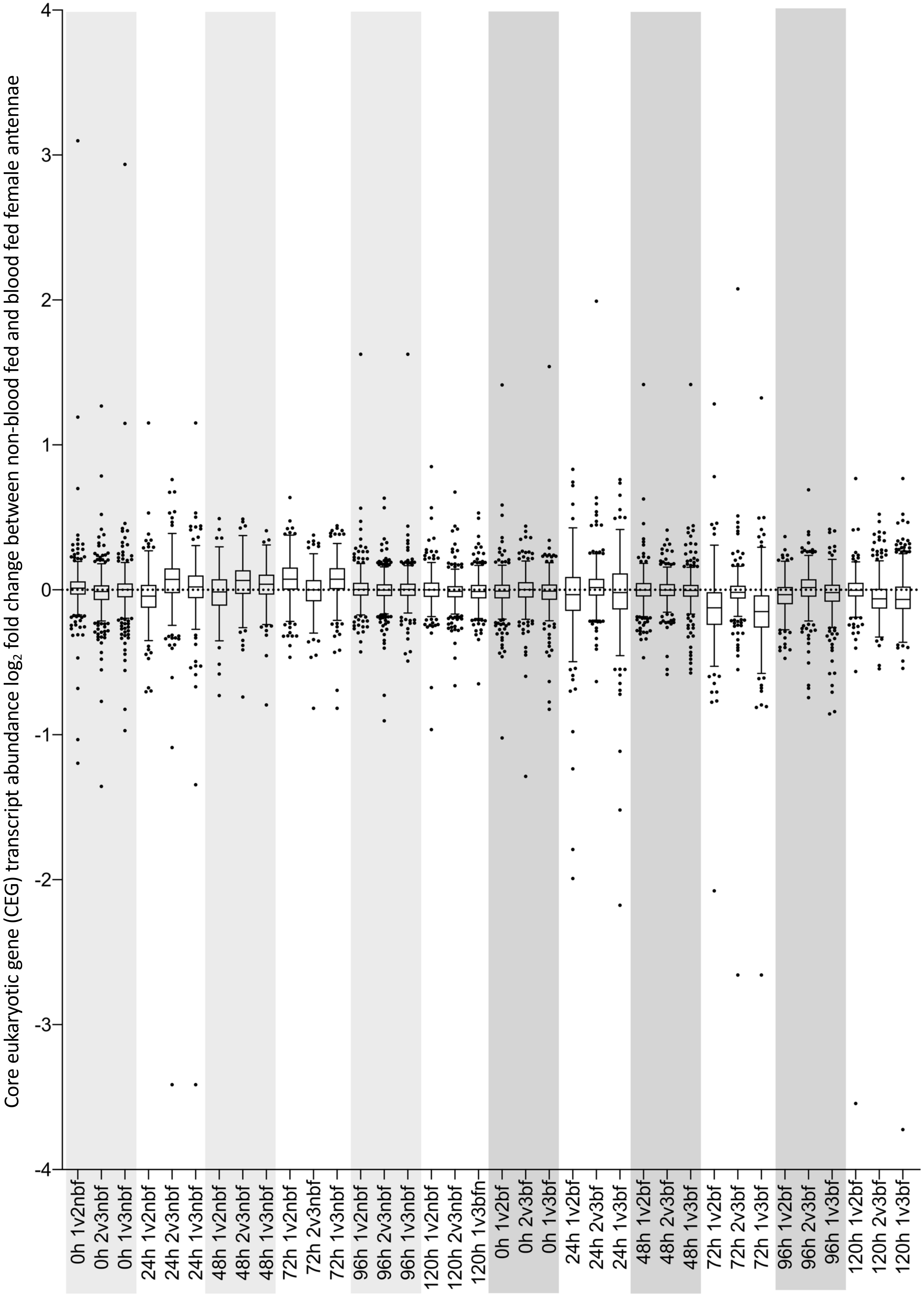
Core eukaryotic gene (CEG) transcript abundance $\log_2$ fold change between non-blood fed and blood fed female antennae
4
3
2
1
0
-1
-2
-3
-4
0h 1v2nbf
0h 2v3nbf
0h 1v3nbf
24h 1v2nbf
24h 2v3nbf
24h 1v3nbf
48h 1v2nbf
48h 2v3nbf
48h 1v3nbf
72h 1v2nbf
72h 2v3nbf
72h 1v3nbf
96h 1v2nbf
96h 2v3nbf
96h 1v3nbf
120h 1v2nbf
120h 2v3nbf
120h 1v3bfn
0h 1v2bf
0h 2v3bf
0h 1v3bf
24h 1v2bf
24h 2v3bf
24h 1v3bf
48h 1v2bf
48h 2v3bf
48h 1v3bf
72h 1v2bf
72h 2v3bf
72h 1v3bf
96h 1v2bf
96h 2v3bf
96h 1v3bf
120h 1v2bf
120h 2v3bf
120h 1v3bf
